# Supplementary material for: Mechanistic Insights into Regulation of the ALC1 Remodeler by the Nucleosome Acidic Patch
Source: Cell Rep. Author manuscript; Available in PMC 2021 Mar 9. (PMC7116876; doi:10.1016/j.celrep.2020.108529)
Supplement: Supplementary Materials [file EMS117809-supplement-Supplementary_Materials.zip › 1-s2.0-S2211124720315187-mmc1.pdf]

Cell Reports, Volume *33*

Supplemental Information

Mechanistic Insights into Regulation of the ALC1 Remodeler by the Nucleosome Acidic Patch

Laura C. Lehmann, Luka Bacic, Graeme Hewitt, Klaus Brackmann, Anton Sabantsev, Guillaume Gaullier, Sofia Pytharopoulou, Gianluca Degliesposti, Hanneke Okkenhaug, Song Tan, Alessandro Costa, J. Mark Skehel, Simon J. Boulton, and Sebastian Deindl

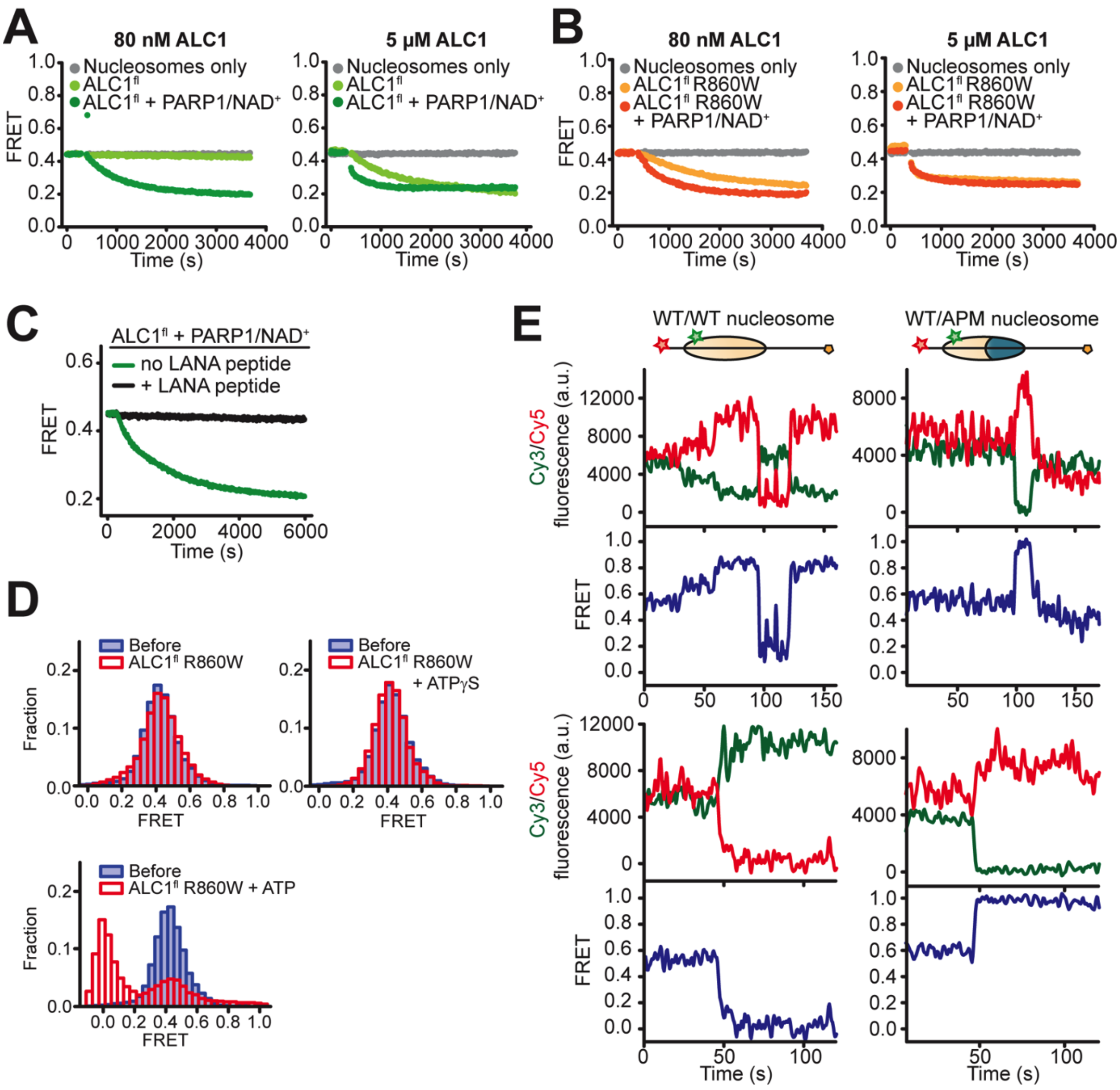

Figure S1. Related to Figure 1. The acidic patch is important for ALC1 remodeling.

(**A**) Ensemble remodeling time courses of initially end-positioned WT/WT nucleosomes (10 nM) alone (grey) or with 80 nM (left) or 5µM (right) ALC1fl with (dark green) or without (light green) 80 nM PARP1 and 50 µM NAD^+. Representative curves from N = 3 independent experiments are shown. (**B**) Ensemble remodeling time courses of initially end-positioned WT/WT nucleosomes (10 nM) alone (grey) or with 80 nM (left) or 5µM (right) ALC1fl R860W with (dark orange) or without (light orange) 80 nM PARP1 and 50 µM NAD^+. Representative curves from N = 3 independent experiments are shown. (**C**) Ensemble remodeling time courses of initially end-positioned WT/WT nucleosomes (10 nM) by 80 nM ALC1fl, activated with 80 nM PARP1 and 50 µM NAD^+, in the presence (black) and absence (dark green) of a peptide derived from the N-terminal portion of the latency associated nuclear antigen (LANA) protein of the Kaposi's sarcoma-associated herpesvirus (KSHV). Representative curves from N = 3 independent experiments are shown. (**D**) Single-molecule FRET histograms for WT/WT nucleosomes before (blue) and after (red) the addition of ALC1fl R860W alone (top left), with ATPγS (top right), or with ATP (bottom). (**E**) Representative donor (green) and acceptor (red) intensity (top), as well as corresponding smFRET time traces (blue, bottom) showing the ALC1fl R860W-catalyzed remodeling of individual WT/WT (left) or WT/APM (right) nucleosomes.

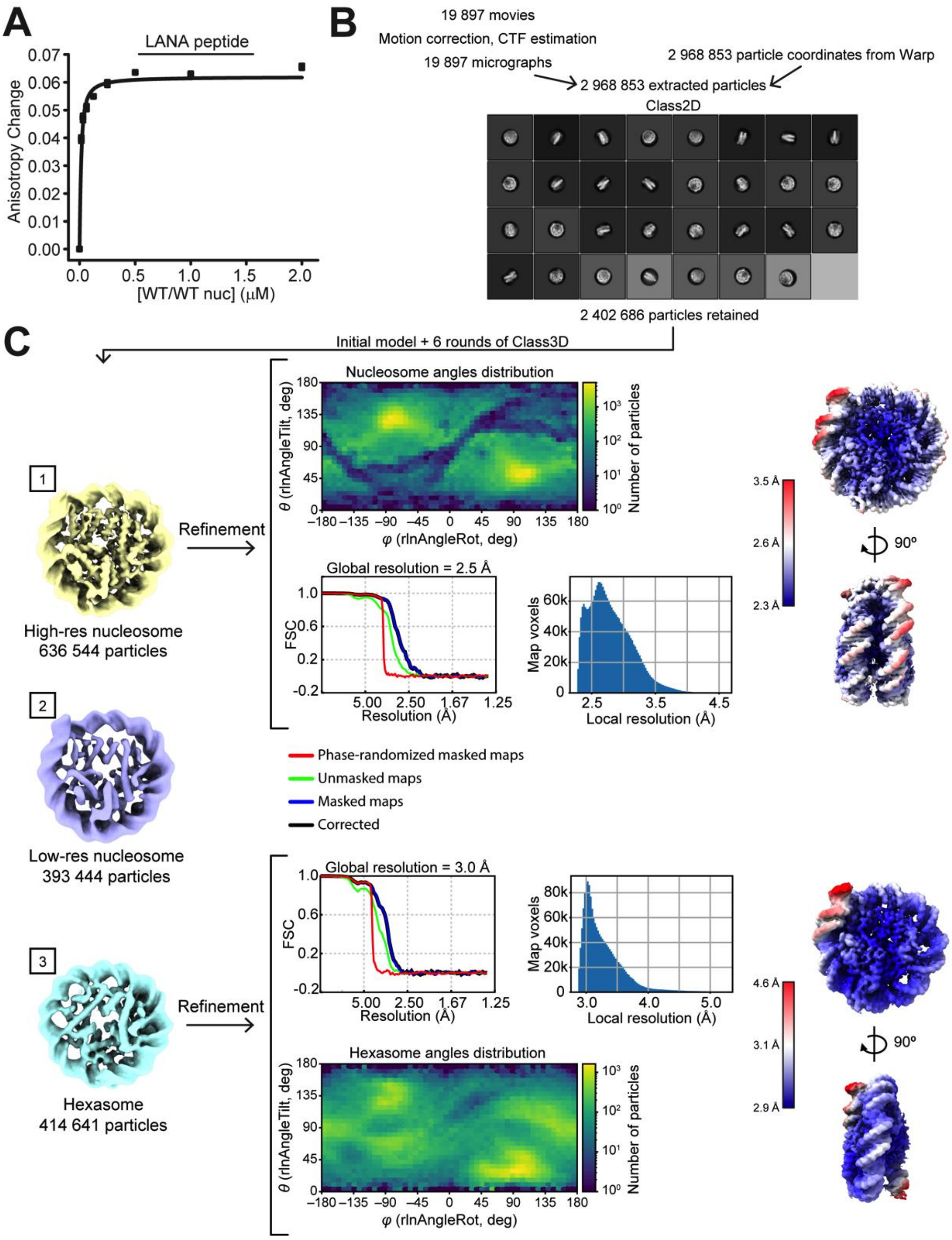

Figure S2. Related to Figure 2. LANA binding to nucleosomes and flowchart for cryo-EM data collection. (**A**) Fluorescence anisotropy binding measurements with TMR-labeled LANA peptide and varying amounts of WT/WT nucleosomes. The dissociation constant (K_d) is 9 nM. Error bars: S.E.M. (N = 3 independent experiments). (**B**) Suitable particles for cryo-EM structure determination were identified by one round of 2D classification followed by three rounds of 3D classification. (**C**) 3D classification identified three classes corresponding to (1) a high-resolution nucleosome, (2) a lower resolution nucleosome, and (3) a hexasome. The high-resolution nucleosome and hexasome classes were further subjected to three more rounds of 3D classification, yielding subsets of homogeneous particles suitable for refinement. Refinement protocol: 3D auto-refinement, three CTF refinements (beam tilt, anisotropic magnification, per-particle defocus and per-micrograph astigmatism), one 3D auto-refinement, Bayesian polishing, and a final 3D auto-refinement.

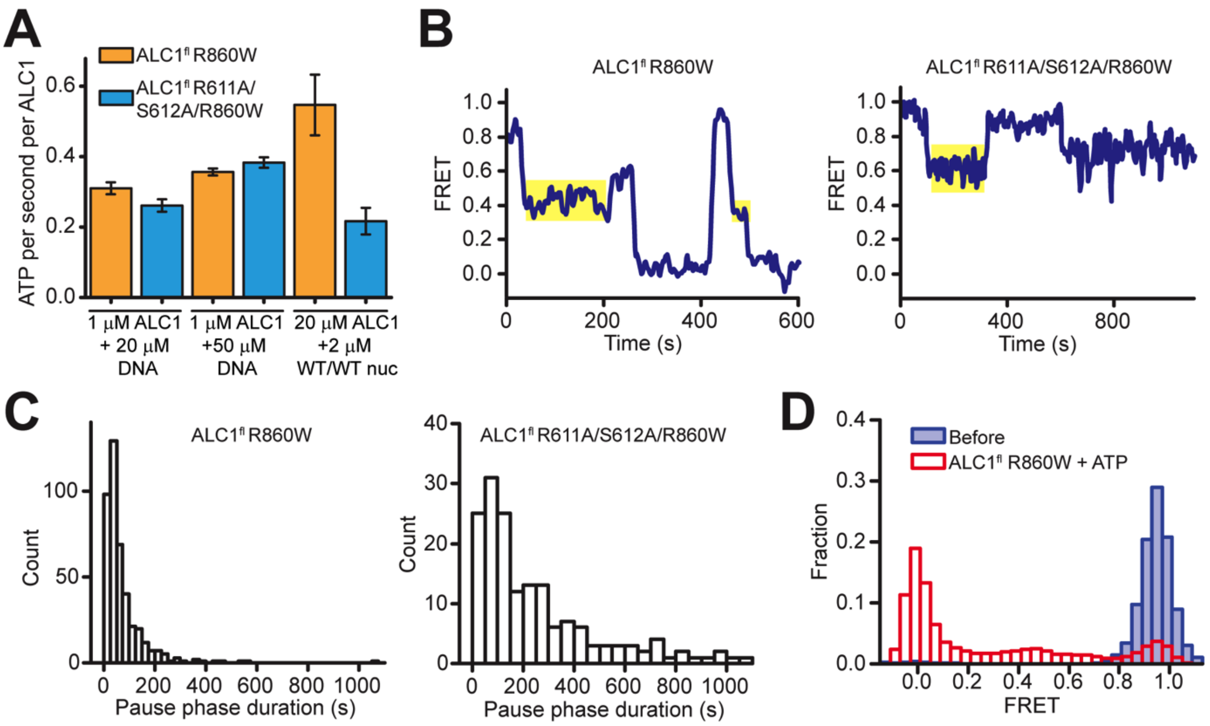

Figure S3. Related to Figure 3. Mutations in the regulatory linker segment of ALC1 affect the pause phase duration during nucleosome remodeling.

(**A**) ATPase rates for 1 µM ALC1^{fl} R860W (orange) or ALC1^{fl} R611A/S612A/R860W (light blue) in the presence of 20 µM or 50 µM dsDNA and 1 mM ATP. For comparison, the ATPase rates for 20 µM ALC1^{fl} R860W or ALC1^{fl} R611A/S612A/R860W in the presence of 2 µM WT/WT nucleosomes and 1 mM ATP are shown (same as shown in Figure 3A). Error bars represent standard deviation (N = 3 independent experiments). (**B**) Representative single-molecule FRET time traces showing the remodeling of individual WT/WT nucleosomes with 3 and 78 bp of flanking linker DNA by ALC1^{fl} R860W (left) and by ALC1^{fl} R611A/S612A/R860W (right). Pause phases are highlighted in yellow. (**C**) Histograms of pause phase durations derived from single-molecule FRET time traces for ALC1^{fl} R860W (left) and ALC1^{fl} R611A/S612A/R860W (right) (N = 421 or 171 plateaus from 166 or 220 time traces for ALC1^{fl} R860W or ALC1^{fl} R611A/S612A/R860W, respectively). (**D**) Single-molecule FRET histograms for WT/WT nucleosomes before (blue) and after (red) the addition of ALC1^{fl} R860W and ATP.

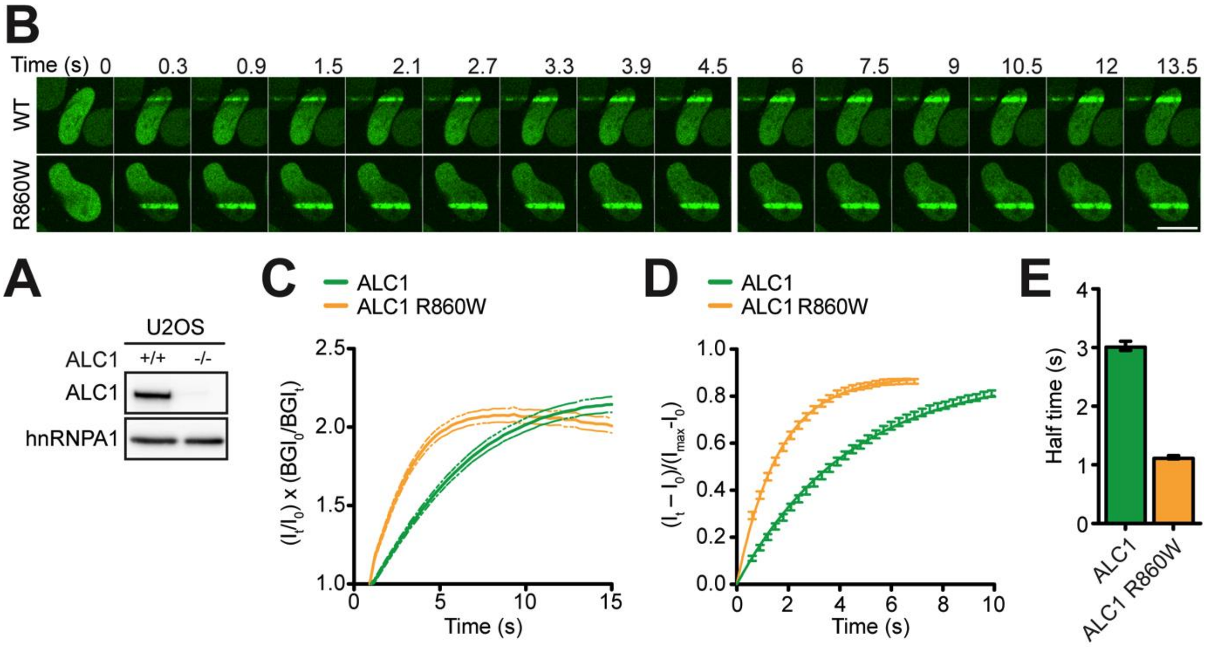

Figure S4. Related to Figure 4. Kinetics of YFP-ALC1 and YFP-ALC1 R860W association with DNA breaks.

(**A**) CRISPR-mediated inactivation of ALC1 in U2OS cells. Immunoblot of whole cell extracts from ALC1$^{+/+}$ and ALC1$^{-/-}$ cells, probed for ALC1. Heterogeneous Nuclear Ribonucleoprotein A1 (hnRNPA1) was probed for as a loading control. (**B)** Representative images of U2OS cells expressing WT or R860W YFP-ALC1, recorded at the specified time points upon laser damage. Scale bar: 10 µm. (**C)** Kinetics of WT (green) and R860W (orange) YFP-ALC1 association with DNA breaks. Fluorescence intensities were normalized and corrected for photobleaching (see Methods section). Error bars represent S.E.M. ($N \geq 131$ traces from 3 independent experiments). (**D**) Fraction of maximal recruitment of WT (green) and R860W (orange) YFP-ALC1 to DNA breaks. Error bars represent S.E.M. ($N \geq 131$ traces from 3 independent experiments). Solid line represents one-phase association. (**E**) Half-time quantified from (D). Data are mean ± 95% confidence intervals.

Protein 1	Position 1	Protein 2	Position 2
ALC1^{fl} R857Q	130	H3	5
ALC1^{fl} R857Q	130	H3	28
ALC1^{fl} R857Q	141	H3	5
ALC1^{fl} R857Q	141	H3	15
ALC1^{fl} R857Q	141	H3	19
ALC1^{fl} R857Q	141	H3	28
ALC1^{fl} R857Q	144	H3	5
ALC1^{fl} R857Q	144	H3	10
ALC1^{fl} R857Q	144	H3	19
ALC1^{fl} R857Q	144	H3	28
ALC1^{fl} R857Q	310	H3	80
ALC1^{fl} R857Q	320	H4	60
ALC1^{fl} R857Q	605	H2B	106
ALC1^{fl} R857Q	605	H2B	113
ALC1^{fl} R857Q	605	H2B	118
ALC1^{fl} R857Q	605	H2B	123
ALC1^{fl} R857Q	605	H4	9
ALC1^{fl} R857Q	607	H2B	123
ALC1^{fl} R857Q	612	H3	28
ALC1^{fl} R857Q	616	H2B	106
ALC1^{fl} R857Q	630	H2A	96
ALC1^{fl} R857Q	630	H2B	106
ALC1^{fl} R857Q	630	H2B	123
ALC1^{fl} R857Q	630	H3	80
ALC1^{fl} R857Q	630	H4	78
ALC1^{fl} R857Q	653	H3	28
ALC1^{fl} R857Q	661	H4	45
ALC1^{fl} R857Q	662	H3	15
ALC1^{fl} R857Q	757	H3	4
ALC1^{fl} R857Q	757	H3	5
ALC1^{fl} R857Q	757	H3	19
ALC1^{fl} R857Q	793	H2A	6
ALC1^{fl} R857Q	793	H3	5
ALC1^{fl} R857Q	793	H3	10
ALC1^{fl} R857Q	826	H3	19
ALC1^{fl} R857Q	848	H2A	96
ALC1^{fl} R857Q	848	H2B	106
ALC1^{fl} R857Q	848	H2B	118
ALC1^{fl} R857Q	848	H3	57

Table S1. Related to Figure 2. List of crosslinks between ALC1^{fl} and histone core proteins.
Cross-links to the ATPase domain of ALC1 (Protein 1) are shown in light green, to the linker region in magenta, and to the macro domain in dark blue. Crosslinks to the core histones (Protein 2) are shown in yellow for H2A, red for H2B, dark green for H3, and light blue for H4.

	Nucleosome class (EMD-11220, PDB 6ZHX)	Hexasome class (EMD-11221, PDB 6ZHY)
**Data collection and processing**		
Acceleration voltage (kV)	300	
Spherical aberration (mm)	2.7	
Amplitude contrast (fraction)	0.1	
Image pixel size (Å/pixel)	0.654	
Electron exposure per frame ($e^-/Å^2$)	0.84	
Number of movie frames	60	
Total electron exposure ($e^-/Å^2$)	50.4	
Nominal defocus range (µm)	-1 to -3	
Number of movies collected	19 897	
Number of picked particles	2 968 853	
Particles used for reconstruction	636 544	414 641
Map symmetry imposed	C1	
Map sharpening B-factor ($Å^2$)	-10	
Global resolution at 0.143 FSC (Å)	2.5	3.0
Local resolution range (Å)	2.3 - 3.5	2.9 - 4.6
**Model building and refinement**		
Initial models used (PDB codes)	3LZ0, 1ZLA	
Number of atoms (hydrogens)	21 581 (9 625)	16 141 (7 186)
Number of protein residues	759	557
Number of DNA residues	290	220
Bond length RMSD (Å)	0.014	0.014
Bond angles RMSD (º)	2.062	2.103
MolProbity score	0.62	0.55
Clash score	0.23	0.12
Ramachandran outliers/allowed/favored (%)	0.00 / 1.62 / 98.38	0.00 / 1.66 / 98.34
Rotamer outliers (%)	1.11	0.65
Cα outliers (%)	0.28	0.58
CαBLAM outliers (%)	0.56	0.76
Model to map CC (mask/peaks/volume)	0.78 / 0.68 / 0.78	0.80 / 0.74 / 0.80

Table S2. Related to Figure 2. Cryo-EM data collection, data processing, model building and model refinement, and validation statistics.

Mutations/ Constructs	Forward Primers	Reverse Primers
H2A E61A /E64A /D90A/E92A	Step 1 GTATCTGACCGCTGCGATTTTGGA ATTGGC Step 2 ACCGCTGCGATTTTGGCGTTGGCC GGGAAT Step 3 AGCTCGCTGTGCGCAACGCGGAG GCACTGAACAAACTGCTCGGAA	Step 1 GCCAATTCCAAAATCGCAGCGGTCA GATAC Step 2 ATTCCCGGCCAACGCCAAAATCGCA GCGGT Step 3 TTCCGAGCAGTTTGTTCAGTGCCTCC GCGTTGCGCACAGCGAGCT
ALC1^{fl} R611A/S612A /R860W	AAAGCGAGCCAGGAAGGTGCGGC GCTGCGTAATAAAGGCTCTG	CAGAGCCTTTATTACGCAGCGCCGC ACCTTCCTGGCTCGCTTT
ALC1^{fl} R611Q/R860W	CGAGCCAGGAAGGTCAGTCTCTG CGTAATAAAG	CTTTATTACGCAGAGACTGACCTTCC TGGCTCG
ALC1 R611A/S612A /R860W	GAAAGCTAGTCAAGAGGGCGCTG CACTCCGAAATAAAGGCAG	CTGCCTTTATTTCGGAGTGCAGCGCC CTCTTGACTAGCTTTC
ALC1 R611Q/R860W	GCCAGGAAGGTCAGTCTCTGCGT AATAAAGGC	GCCTTTATTACGCAGAGACTGACCT TCCTGGC
63-601-0-Cy5 nucl. DNA	CGCTGTTTTCGAATTTACCC	/5Cy5/ATCAGAATCCCGGTGCCG
63-601-0 nucl. DNA	CGCTGTTTTCGAATTTACCC	ATCAGAATCCCGGTGCCG
0-601-0 nucl. DNA	ATCGATGTATATATCTGACACGTG CC	ATCAGAATCCCGGTGCCG

Table S3. Related to STAR Methods. List of DNA oligonucleotides that were used in this study.

Methods S1. Related to STAR Methods. Details on the structural modeling.

The ALC1-linker peptide used to form a complex with the nucleosome for structure determination by cryo-EM has the following sequence:

```
EKASQ E  G  R  S  LRNKGSVLIPGLVEGSTKRKRVLSPEEK-Biotin
     -2 -1  0 +1 +2
```

The experimental density in our cryo-EM map can accommodate four residues (Figure M1, Figure M2), with a well-defined arginine side chain density ('Arg anchor') being the most salient feature of the map.

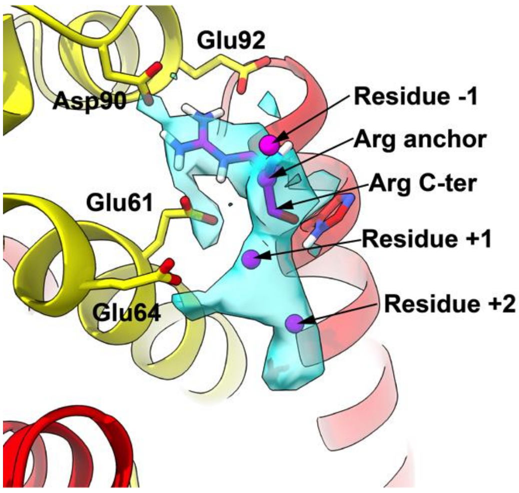

Figure M1. Arg anchor residue (sticks) and Cα atoms of one residue upstream and two residues downstream (spheres).

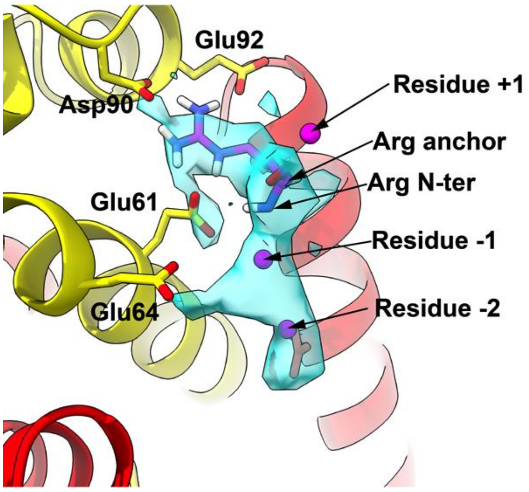

Figure M2. Arg anchor residue (sticks) and Cα atoms of two residues upstream and one residue downstream (spheres).

Since the ALC1-linker peptide contains three other arginine residues in addition to R611, we considered all of them when building our model (Figure M1):

Segment A: GRSL (highlighted in green in the sequence above)
Segment B: LRNK (underlined, overlapping with Segment A in green)
Segment C: KRKR (highlighted in red)
Segment D: KRVL (underlined, overlapping with Segment C in red)

In addition to these four segments with the arginine anchor at position 2, we also examined the following 4 segments with the arginine anchor at position 3, corresponding to the four segments above but modeled in the opposite N-terminal to C-terminal direction (Figure M2):

Segment E: EGRS
Segment F: SLRN
Segment G: TKRK
Segment H: RKRV

We built each of these eight four-residue segments and individually subjected them to real-space refinement against the cryo-EM map using the molecular dynamics flexible fitting (MDFF) engine implemented in ISOLDE (Croll, 2018). This approach not only aims to optimize the fit of the model to the map, but also imposes physically allowed conformations and electrostatic interactions in the model, with an automatically determined weighting factor between the map and the molecular dynamics force field. Therefore, the resulting

model is necessarily a compromise between these two optimization targets, and an optimal model may not exhibit a perfect fit to the map, since all cryo-EM maps intrinsically feature experimental measurement errors. Importantly, the resulting model features energetically and sterically allowed conformations.

The figures below show the result of such real-space refinement for each of the eight four-residue segments and follow the same color code as the one used in the manuscript: yellow for histone H2A, red for histone H2B, magenta for the ALC1-linker and translucent blue for the experimental cryo-EM density. In all figure legends, the sequence of each segment is indicated and the arginine residue occupying the “anchor” position is underlined.

Segment C (K<u>R</u>KR) does not fit the density, with a lysine residue too bulky to be accommodated without steric clashes in the space between the peptide backbone and residue Glu64 of H2A (Figure M3). Although this lysine was built pointing straight at H2A-Glu64, MDFF promptly moved it away. Modeling this segment with the opposite N-terminal to C-terminal polarity (Segment G) also places a lysine at this location, and therefore results in a similarly poor fit to the map (Figure M4).

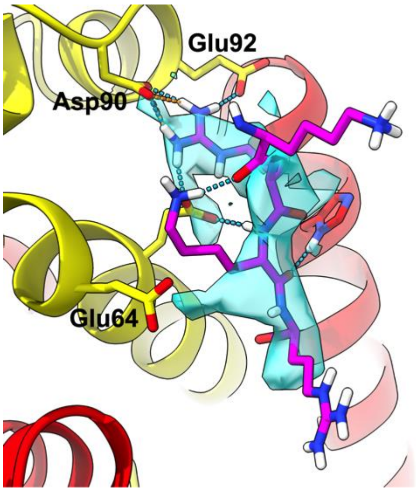

Figure M3. Segment C: K<u>R</u>KR

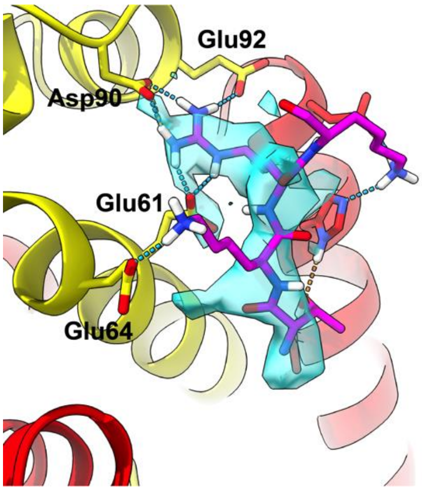

Figure M4. Segment G: TK<u>R</u>K

Segment D (K<u>R</u>VL) does not fit the density and does not make sense chemically with the hydrophobic valine residue facing the charged residue Glu64 of H2A, which is less favored than a hydrogen-bond donor (Figure M5). Modeling the opposite N-terminal to C-terminal polarity (Segment H) places a lysine at this location which, as we showed in Figure M3 and M4 above, does not fit the density either (Figure M6).

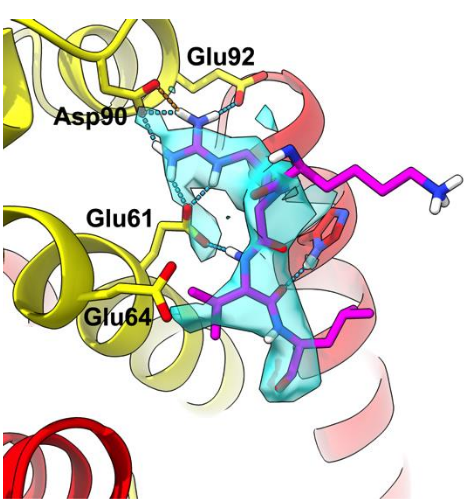

Figure M5. Segment D: K<u>R</u>VL

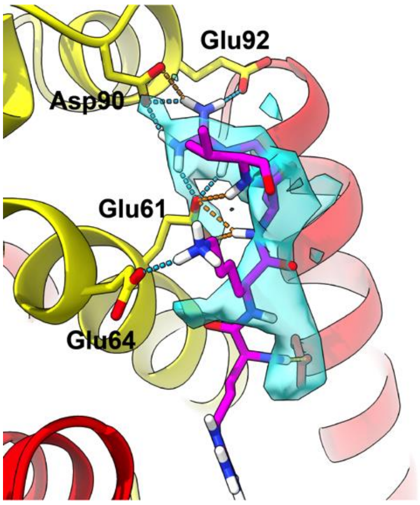

Figure M6. Segment H: RK<u>R</u>V

Segment B (LRNK) has an asparagine residue able to establish a hydrogen-bond with residue Glu64 of H2A (Figure M7), but this residue does not fit the density as well as the serine residue in Segment A (GRSL, Figure M9; see also Table M1). Modeling the opposite N-terminal to C-terminal polarity (Segment F) places a leucine in front of residue Glu64 of H2A, which is too bulky to be accommodated in this space and also does not make sense chemically since it cannot establish a hydrogen bond with H2A-Glu64 which, as a result, gets pushed away and no longer satisfactorily explains the density at its location (Figure M8).

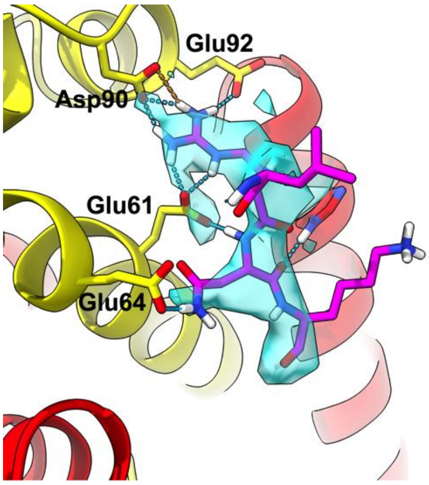

Figure M7. Segment B: LRNK

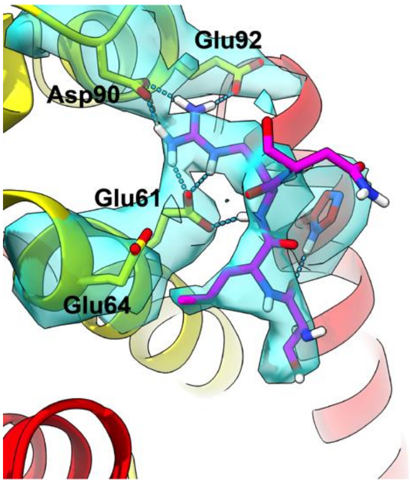

Figure M8. Segment F: SLRN
The map is displayed at the same contour level as in other figures, but also displayed around histone residues to show the poor fit of H2A-Glu64.

Segment A (GRSL) explains the observed density better than all the models shown above and establishes a more favorable hydrogen-bond network with histones than most of the other models (Figure M9). Modeling the opposite N-terminal to C-terminal polarity (Segment E) places a glycine in front of residue Glu64 of H2A, which does not explain the observed density, since glycine has no side chain (Figure M10), and leaves Glu64 of H2A without a hydrogen-bond donor.

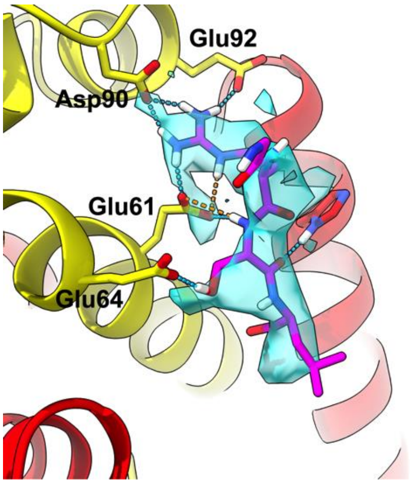

Figure M9. Segment A: GRSL

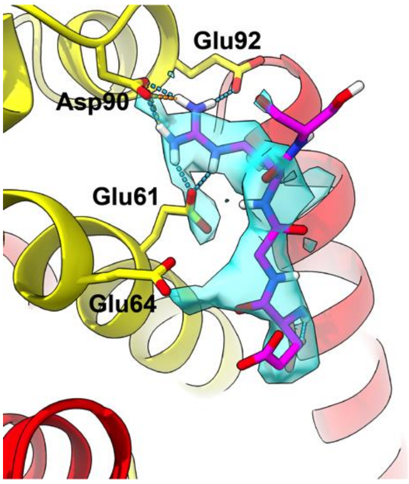

Figure M10. Segment E: EGRS

Clearly, all models with an N-terminal to C-terminal directionality opposite to Segment A display extremely poor fits to the map (Figures M4, M6, M8 and M10). Among the models with correct N-terminal to C-terminal directionality (Segments A-D), Segment A (GRLS) is the one that best explains the observed density while also fulfilling chemical constraints with a sensible hydrogen-bonding network and electrostatic interactions.

To obtain an unbiased, quantitative measure of how well each model with correct N-terminal to C-terminal directionality fits the map, we calculated per-residue real-space correlation coefficients (RSCC) between each model and the map, using the program phenix.validation_cryoem from the Phenix suite (Liebschner et al., 2019). The results are summarized in Table M1 below. RSCC rows are duplicated because density assigned to the ALC1 linker is present on both acidic patches of the nucleosome, and we modeled the peptide and analyzed the model fit into the density on both sides of the nucleosome (all figures above show chain K).

**Segment A sequence**	**G**	**R**	**S**	**L**
Segment A RSCC (chain K)	0.518	0.562	0.574	0.501
Segment A RSCC (chain L)	0.411	0.551	0.544	0.465
**Segment B sequence**	**L**	**R**	**N**	**K**
Segment B RSCC (chain K)	0.458	0.586	0.487	0.388
Segment B RSCC (chain L)	0.485	0.567	0.442	0.434
**Segment C sequence**	**K**	**R**	**K**	**R**
Segment C RSCC (chain K)	0.342	0.535	0.425	0.433
Segment C RSCC (chain L)	0.470	0.554	0.401	0.431
**Segment D sequence**	**K**	**R**	**V**	**L**
Segment D RSCC (chain K)	0.352	0.565	0.451	0.393
Segment D RSCC (chain L)	0.403	0.536	0.426	0.463

Table M1. RSSC values for each of the four residues of the ALC1 linker in each of the models with correct N-terminal to C-terminal directionality. The arginine residue occupying the "anchor" position is underlined.

While the arginine residue inserted into the acidic patch binding pocket exhibits high RSCC values regardless of the identity of the surrounding residues in the model (Table M1, column highlighted in dark grey), the model with a serine residue at the C-terminus of this arginine anchor shows a markedly higher RSCC at this location in

comparison to all the other models (Table M1, column highlighted in light grey). By this objective metric, Segment 1 (GRSL, as initially presented in our manuscript, highlighted in green in Table M1) is the one that best explains the observed density over all four residues spanned by the additional density.

To summarize, we modeled Segment A (GRSL) because it clearly best explains the observed density, both in terms of visual inspection of the experimental cryo-EM map and also quantitatively in terms of the per-residue real-space correlation coefficients.
