# Supplementary material for: Mechanistic Insights into Regulation of the ALC1 Remodeler by the Nucleosome Acidic Patch
Source: Cell Rep. Author manuscript; Available in PMC 2021 Mar 9. (PMC7116876; doi:10.1016/j.celrep.2020.108529)
Supplement: Supplementary Materials [file EMS117809-supplement-Supplementary_Materials.zip › 1-s2.0-S2211124720315187-mmc2.pdf]

Cell Reports

Report

Mechanistic Insights into Regulation of the ALC1 Remodeler by the Nucleosome Acidic Patch

Graphical Abstract

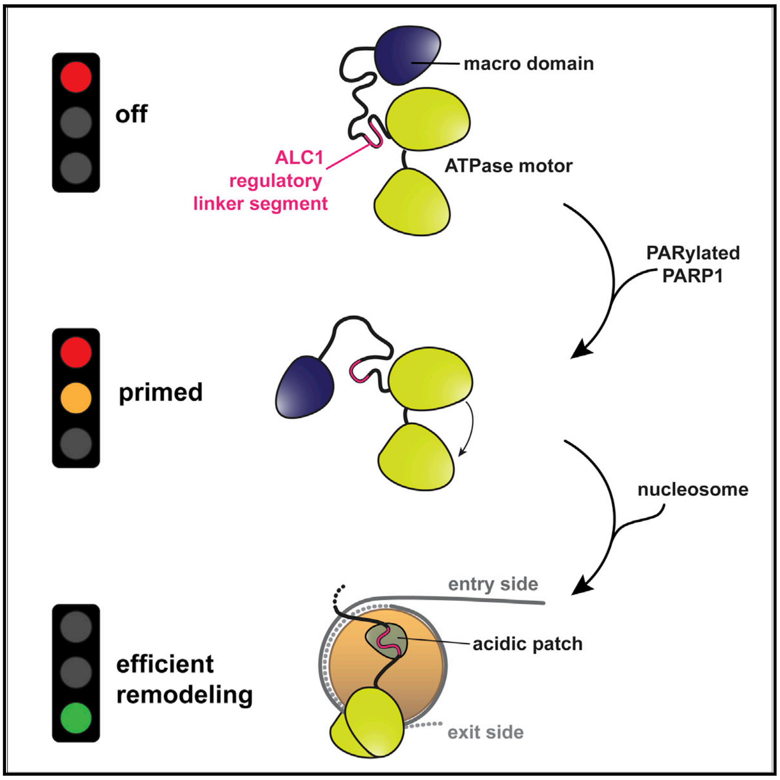

Highlights

- Remodeling by ALC1 requires an acidic patch on the nucleosome entry side
- A cryo-EM structure reveals how a regulatory linker segment binds to the acidic patch
- Interfacial mutations compromise recruitment to DNA damage and catalytic activities

Authors

Laura C. Lehmann, Luka Bacic, Graeme Hewitt, ..., J. Mark Skehel, Simon J. Boulton, Sebastian Deindl

Correspondence

simon.boulton@crick.ac.uk (S.J.B.), sebastian.deindl@icm.uu.se (S.D.)

In Brief

Lehmann et al. show that full activation of the ALC1 remodeler requires interactions between its regulatory linker segment and the acidic patch of the nucleosome, which tether the remodeler to the nucleosome and efficiently couple remodeler-catalyzed ATP hydrolysis to nucleosome mobilization.

Lehmann et al., 2020, Cell Reports *33*, 108529
December 22, 2020 © 2020 The Author(s).
https://doi.org/10.1016/j.celrep.2020.108529

Cell Reports

CellPress
OPEN ACCESS

Report

Mechanistic Insights into Regulation of the ALC1 Remodeler by the Nucleosome Acidic Patch

Laura C. Lehmann,[1,7] Luka Bacic,[1,7] Graeme Hewitt,[2,7] Klaus Brackmann,[1] Anton Sabantsev,[1] Guillaume Gaullier,[1] Sofia Pytharopoulou,[1] Gianluca Degliesposti,[3] Hanneke Okkenhaug,[4] Song Tan,[5] Alessandro Costa,[6] J. Mark Skehel,[3] Simon J. Boulton,[2,*] and Sebastian Deindl[1,8,*]
[1]Department of Cell and Molecular Biology, Science for Life Laboratory, Uppsala University, 75124 Uppsala, Sweden
[2]The Francis Crick Institute, 1 Midland Road, London NW1 1AT, UK
[3]MRC Laboratory of Molecular Biology, Francis Crick Avenue, Cambridge Biomedical Campus, Cambridge CB2 0QH, UK
[4]Imaging Facility, Babraham Institute, Cambridge CB22 3AT, UK
[5]Center for Eukaryotic Gene Regulation, Department of Biochemistry and Molecular Biology, The Pennsylvania State University, University Park, PA 16802, USA
[6]Macromolecular Machines Laboratory, The Francis Crick Institute, 1 Midland Road, London NW1 1AT, UK
[7]These authors contributed equally
[8]Lead contact
*Correspondence: simon.boulton@crick.ac.uk (S.J.B.), sebastian.deindl@icm.uu.se (S.D.)
https://doi.org/10.1016/j.celrep.2020.108529

SUMMARY

Upon DNA damage, the ALC1/CHD1L nucleosome remodeling enzyme (remodeler) is activated by binding to poly(ADP-ribose). How activated ALC1 recognizes the nucleosome, as well as how this recognition is coupled to remodeling, is unknown. Here, we show that remodeling by ALC1 requires a wild-type acidic patch on the entry side of the nucleosome. The cryo-electron microscopy structure of a nucleosome-ALC1 linker complex reveals a regulatory linker segment that binds to the acidic patch. Mutations within this interface alter the dynamics of ALC1 recruitment to DNA damage and impede the ATPase and remodeling activities of ALC1. Full activation requires acidic patch-linker segment interactions that tether the remodeler to the nucleosome and couple ATP hydrolysis to nucleosome mobilization. Upon DNA damage, such a requirement may be used to modulate ALC1 activity via changes in the nucleosome acidic patches.

INTRODUCTION

Packaging into chromatin creates a barrier to DNA transactions (Lorch and Kornberg, 2017). The repeat unit of chromatin, the nucleosome, contains ~150 base pairs (bp) of DNA wrapped around a histone octamer (Luger et al., 1997). ATP-dependent nucleosome remodeling enzymes (remodelers) feature a conserved Snf2 (sucrose non-fermenter 2) family ATPase (Flaus et al., 2006) and play vital roles in regulating accessibility to DNA (Bartholomew, 2014; Becker and Workman, 2013; Bowman, 2010; Clapier et al., 2017; Narlikar et al., 2013; Lusser and Kadonaga, 2003).

Many proteins interact with a negatively charged "acidic patch" (AP), formed by histones H2A and H2B on both faces of the nucleosome (Figure 1A), by inserting an arginine (Arg) side chain into a pocket defined predominantly by H2A E61, E64, D90, and E92 (McGinty and Tan, 2016; Zhou et al., 2019). Previous studies have identified interactions between the AP and several remodelers, including SWR1 (Willhoft et al., 2018), INO80 (Eustermann et al., 2018; Ayala et al., 2018), BAF (He et al., 2020), and RSC (Valencia et al., 2019; Wagner et al., 2020; Ye et al., 2019). These structures suggested that the remodeler-AP interaction acts as a physical tether in the assembly of the nucleosome-remodeler complex. Moreover, disrupting the AP interaction by the deletion of entire remodeler subunits impaired remodeling (Eustermann et al., 2018; Willhoft et al., 2018). Recently, the AP has been implicated in the activation of imitation-switch (ISWI)-family remodelers and chromodomain helicase DNA-binding protein 1 (Chd1) (Dann et al., 2017; Dao et al., 2020; Gamarra et al., 2018; Goldman et al., 2010; Levendosky and Bowman, 2019), and an AP-interacting basic motif was shown to be essential for remodeling by the ISWI remodeler SNF2h (Dao et al., 2020).

The ALC1 remodeler (Amplified in liver cancer 1) is encoded on a chromosome region frequently amplified in hepatocarcinomas (Ma et al., 2008; Marchio et al., 1997; Wong et al., 2003). ALC1 has been implicated in DNA damage response (Ahel et al., 2009; Chen et al., 2009; Gottschalk et al., 2009, 2012; Pines et al., 2012; Sellou et al., 2016) and oncogenesis (Chen et al., 2010, 2009; Ma et al., 2008; Marchio et al., 1997; Mu et al., 2015; Pines et al., 2012; Sellou et al., 2016) and differs from other remodelers by virtue of its macro domain, which binds poly(ADP-ribose) (PAR) chains (Ahel et al., 2009; Gottschalk et al., 2009) at sites of DNA damage (Hassa et al., 2006; Lindahl et al., 1995; Satoh and Lindahl, 1992). PARylation is linked with chromatin relaxation (Frechette et al., 1985; Leduc et al., 1986; de Murcia et al., 1986; Poirier et al., 1982), which is thought to promote DNA repair (D'Amours et al., 1999; El-Khamisy et al., 2003; Malanga

This is an open access article under the CC BY license (http://creativecommons.org/licenses/by/4.0/).

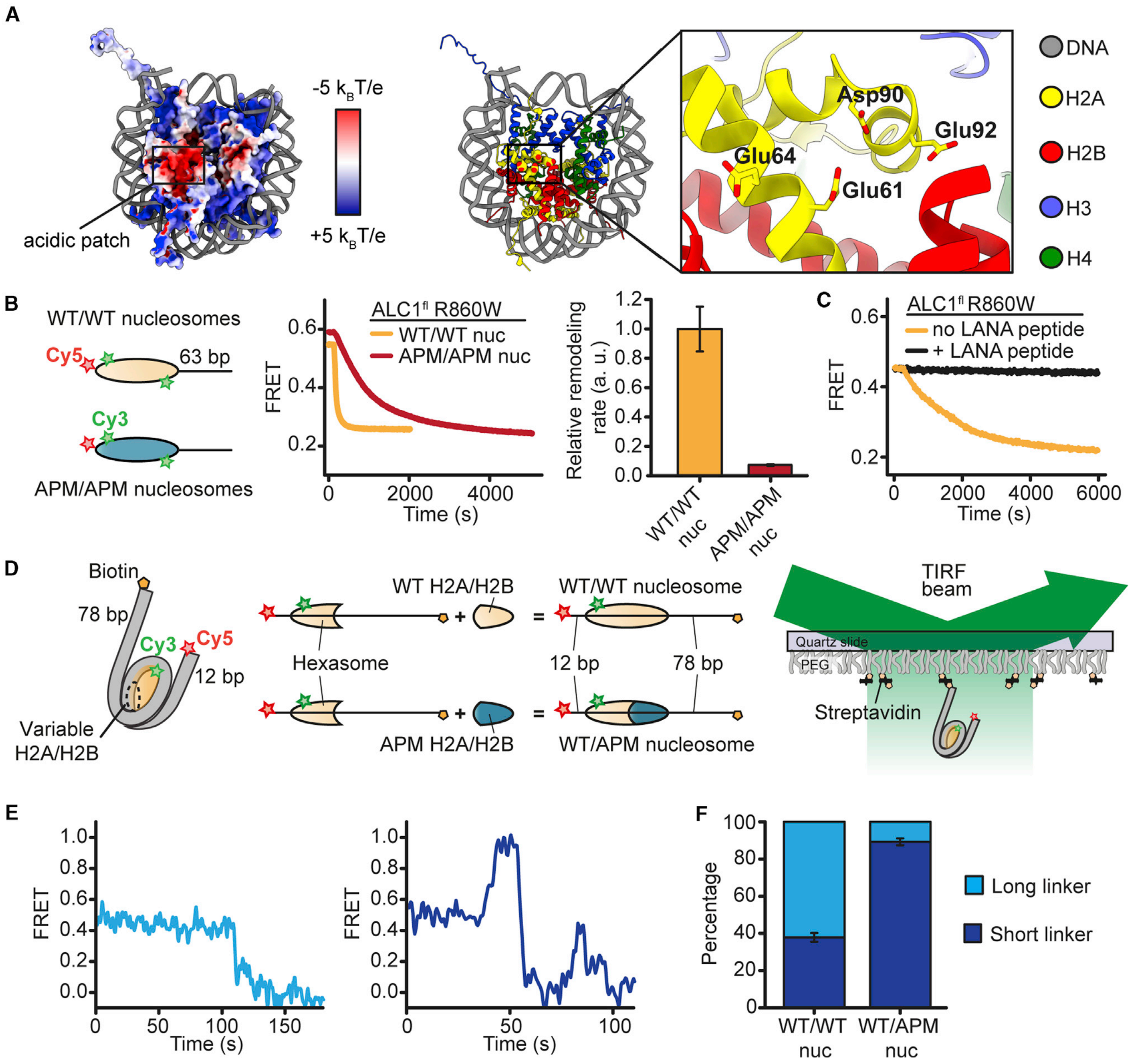

Figure 1. The AP Is Important for Remodeling by ALC1

(A) Left: octamer surface colored by electrostatic potential (from −5 k_B T/e, indicated in red, to +5 k_B T/e, indicated in blue). Right: key residues of the H2A AP. Based on PDB: 1AOI.

(B) Left: schematic of FRET-labeled wild-type (WT/WT) nucleosomes and APM/APM nucleosomes with AP mutations on both faces of the octamer. Middle: ensemble remodeling of WT/WT and APM/APM nucleosomes (10 nM) in the presence of 20 μM ALC1^{fl} R860W. Right: relative remodeling rates. Error bars represent SD (n = 3 independent experiments).

(C) Ensemble remodeling of WT/WT nucleosomes (10 nM) by 80 nM ALC1^{fl} R860W with or without LANA peptide.

(D) smFRET labeling and detection.

(E) ALC1^{fl} R860W-catalyzed remodeling of individual WT/WT nucleosomes toward the longer (left) or shorter (right) linker DNA.

(F) Percentages of traces with the initial remodeling direction toward longer or shorter linker DNA for WT/WT and WT/APM nucleosomes. Error bars indicate SEM (n > 100 traces).

See also Figure S1.

and Althaus, 2005; Realini and Althaus, 1992). Initial chromatin relaxation involves PAR polymerase 1 (PARP1) and ALC1 and enables the PAR-dependent recruitment of additional proteins (Smith et al., 2018). Early PAR-dependent remodeling promotes DNA exposure but does not affect histone accessibility (Smith et al., 2019).

In the absence of DNA damage, the juxtaposition of the macro domain of ALC1 against its ATPase maintains an inactive conformation (Lehmann et al., 2017; Singh et al., 2017). PAR binding to the macro domain relieves this autoinhibition upon recruitment to DNA damage (Lehmann et al., 2017; Singh et al., 2017). However, the mechanism by which ALC1 recognizes the nucleosome, and how that recognition is coupled to remodeling, is unknown.

Here, we establish that nucleosome repositioning by ALC1 requires a wild-type (WT) AP on the entry side of the nucleosome. Our cryo-electron microscopy (cryo-EM) structure of a nucleosome-ALC1 linker complex reveals a regulatory segment that engages the AP via an Arg anchor. Mutation of this segment or of the AP perturbs ATPase and remodeling activities of ALC1. The regulatory segment thus activates remodeling by ALC1 upon recognition of its *bona fide* nucleosome substrate with intact AP. We anticipate that other remodelers may use a similar mechanism to regulate their activities by analogous interactions between the AP and family-specific regulatory sequence motifs.

RESULTS

Nucleosome Remodeling by ALC1 Requires the Entry-Side AP

To probe whether the nucleosome AP (Figure 1A) might regulate ALC1, we constructed end-positioned nucleosomes with AP mutations (APMs; alanine substitutions on H2A E61A, E64A, D90A, and E92A) on both faces of the octamer (APM/APM nucleosomes). We also reconstituted end-positioned WT nucleosomes with intact APs (WT/WT). We detected nucleosome repositioning by a constitutively active mutant of ALC1, ALC1^{fl} R860W (Lehmann et al., 2017; Figures S1A and S1B), using fluorescence resonance energy transfer (FRET) between a Cy3 donor on H2A and a Cy5 acceptor on the octamer-proximal DNA end (Blosser et al., 2009; Deindl et al., 2013) (Figure 1B). To compare the remodeling of WT/WT and APM/APM nucleosomes under saturating concentrations of ALC1^{fl} R860W and ATP, we first used an ensemble FRET assay (Yang et al., 2006). Upon addition of ALC1^{fl} R860W and ATP to WT/WT nucleosomes, FRET decreased, consistent with DNA moving toward the shorter linker side. The remodeling rate decreased substantially (~13.7-fold) for APM/APM nucleosomes (Figure 1B), indicating an important role of the AP in ALC1-induced remodeling. We next determined the remodeling rate for WT/WT nucleosomes in the presence of sub-saturating concentrations of ALC1^{fl} R860W and saturating concentrations of a peptide derived from the latency-associated nuclear antigen (LANA) of Kaposi's sarcoma-associated herpesvirus. This LANA peptide binds to the AP of the nucleosome (Barbera et al., 2006) and has been used as a competitor to establish AP binding (England et al., 2010; Gamarra et al., 2018). Strikingly, the LANA peptide completely abrogated ALC1-catalyzed remodeling (Figures 1C and S1C).

To examine how ALC1 responds to AP asymmetry, we produced asymmetric nucleosomes with distinct H2A/H2B dimers (Levendosky et al., 2016). We first reconstituted FRET-labeled hexasomes with 12 bp of linker DNA on one side and 78 bp on the other side of the histone core (Figure 1D). The single H2A/H2B dimer in these hexasomes was homogeneously incorporated on the more bendable side of the asymmetric positioning sequence (Levendosky et al., 2016; Lowary and Widom, 1998; Ngo et al., 2015), the shorter linker side. We then combined these oriented hexasomes with WT or APM H2A/H2B dimer to produce either WT/WT or WT/APM nucleosomes (Figure 1D). These nucleosomes exhibited an intermediate starting FRET value (~0.4), providing dynamic range for movements in both directions. To monitor ALC1-catalyzed remodeling of individual WT/WT and WT/APM nucleosomes using single-molecule FRET (smFRET) (Blosser et al., 2009; Deindl and Zhuang, 2012; Deindl et al., 2013), we immobilized them and detected their fluorescence emissions (Figure 1D). Upon addition of ALC1^{fl} R860W and ATP (but not ATPγS; Figure S1D) to WT/WT nucleosomes, a large fraction (62%) of single-nucleosome remodeling traces featured an initial decrease in FRET, consistent with the ability of ALC1 to mobilize the octamer toward the longer linker (Figures 1E, 1F, and S1E). A smaller fraction (38%) displayed the opposite directionality, consistent with Cy5 on the shorter linker moving closer to the octamer. Our data, therefore, indicate a modest preference of ALC1 to center the histone octamer by moving it toward longer linker DNA. In stark contrast, WT/APM nucleosomes were almost exclusively (89%) moved toward the shorter linker end (Figures 1F and S1E), implying that nucleosome repositioning by ALC1 critically depends on an entry-side WT AP.

A Regulatory Segment of the ALC1 Linker Binds to the AP

To probe ALC1-histone interactions, we carried out cross-linking coupled to mass spectrometry (XL-MS). A substantial fraction of the cross-links observed between histones and ALC1 involved its linker region (residues 570–703) (Figure 2A; Table S1). Consistent with an as yet undefined functional role of the linker region, a truncated fragment of ALC1 lacking ~60 amino acids of the linker in addition to the macro domain lost the ability to decompact a LacO array *in vivo* (Singh et al., 2017).

We next aligned the sequence of the LANA peptide (Barbera et al., 2006) to the linker of ALC1 (Figure 2B). Strikingly, an N-terminal regulatory linker segment (ALC1^{RLS}, residues 604–624) exhibited sequence similarity with LANA, including the conserved R611 that aligns with the Arg anchor of LANA (Figure 2B). To directly test whether ALC1^{RLS} binds to the nucleosome, we monitored the fluorescence anisotropy of a tetramethylrhodamine-labeled ALC1^{RLS} peptide (ALC1^{RLS}-TAMRA) in the presence of increasing concentrations of WT/WT nucleosomes. WT/WT nucleosomes exhibited specific affinity for the ALC1^{RLS}-TAMRA peptide (Figure 2C; dissociation constant [K_D], ~1.1 μM). Conversely, nucleosomes with AP mutations on both H2A/H2B dimers displayed substantially reduced affinity for the ALC1^{RLS}-TAMRA peptide (Figure 2C), consistent with its binding to the WT/WT AP. Next, we probed whether the LANA peptide could compete with the ALC1^{RLS} peptide for binding to the nucleosome (Figure 2D). Indeed, the addition of unlabeled ALC1^{RLS} peptide to a pre-formed LANA-TAMRA peptide-nucleosome complex caused a decrease in its fluorescence anisotropy in a dose-dependent manner (Figure 2D; see also Figure S2A), demonstrating that ALC1^{RLS} interacts with the AP.

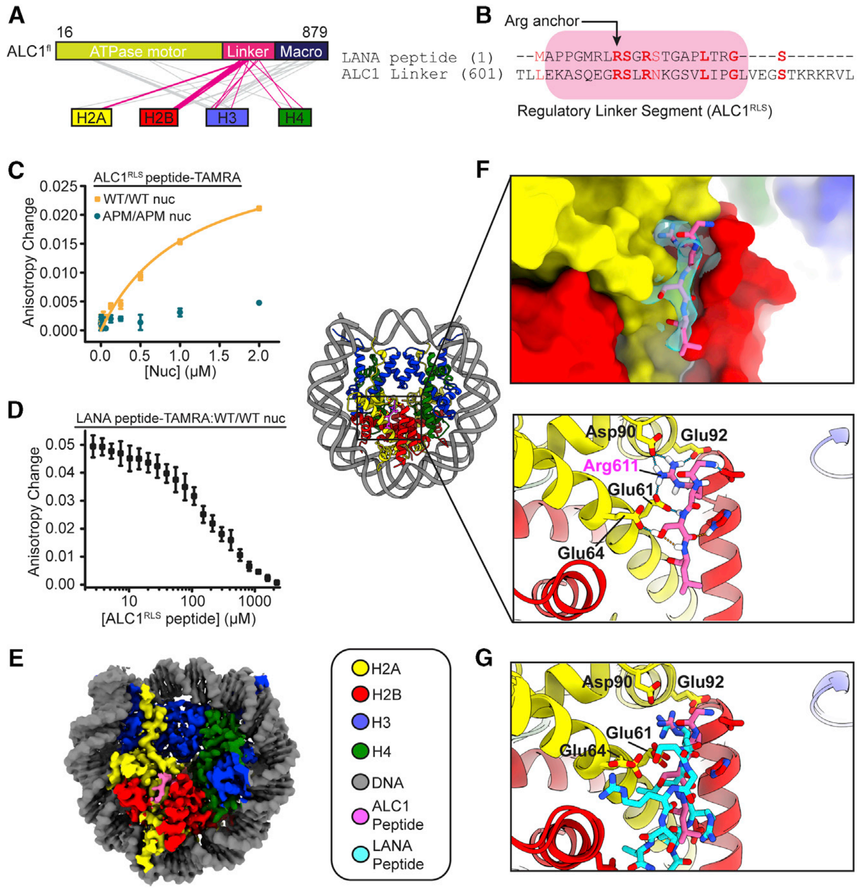

Figure 2. The ALC1 Linker Region Binds to the AP

(A) ALC1^{fl}-histone cross-links. Magenta lines indicate linker-histone cross-links: gray lines indicate other cross-links.

(B) Sequence alignment of LANA (1–22) and ALC1 linker (601–635).

(C) Fluorescence anisotropy of TAMRA-labeled ALC1^{RLS} in the presence of various amounts of WT/WT or APM/APM nucleosomes. Error bars for WT/WT nucleosome curve represent SEM (n = 3 independent experiments). APM/APM nucleosomes: one representative curve out of two independent experiments is indicated. Error bars represent SD (n = 10 technical replicates).

(D) Competition between TAMRA-labeled LANA peptide pre-bound to WT/WT nucleosomes and unlabeled ALC1^{RLS}. See also Figure S2A. Error bars represent SEM (n = 3 independent experiments).

(E) Cryo-EM map.

(F) Left: cartoon representation. Top right inset: cryo-EM density around the ALC1 linker (cyan). Bottom right inset: hydrogen-bonding interactions.

(G) Superposition of PDB: 1ZLA onto the linker-nucleosome complex.

See also Figure S2 and Tables S1 and S2.

Cryo-EM Structure of ALC1^{RLS} Bound to the Nucleosome

We next determined a cryo-EM structure of the cross-linked complex between a linker peptide and the nucleosome (Figures 2E–2G, S2B, and S2C). The map exhibited an overall resolution of 2.5 Å (Figure S2C) with visible side chain features for central residues of ALC1^{RLS} (Figure 2F; mean local resolution around the linker peptide: 2.5 Å). With an overall root-mean-square deviation (RMSD) of 0.46 Å between 739 Cα pairs when compared with the accession number PDB: 1ZLA, the nucleosome in our model is largely undisturbed relative to previous structures (Barbera et al., 2006). The ALC1^{RLS}-AP interaction is stabilized by nine hydrogen bonds as well as van der Waals interactions, and ALC1^{RLS} inserts residue R611 into a pocket formed by the α1-α2 helices of H2A (Figure 2F). Overall, ALC1^{RLS} engages the AP in a manner that bears marked similarity to the binding mode observed for LANA (Barbera et al., 2006), albeit with reverse N- to C-terminal polarity (Figure 2G; RMSD of 1.78 Å between 11 atom pairs).

ALC1^{RLS} Plays a Crucial Role in Activating ATP Hydrolysis and Remodeling

In order to disrupt the AP-ALC1^{RLS} interaction, we mutated the ALC1^{RLS} Arg anchor (R611) and the adjacent residue (S612) to alanines. ALC1^{fl} R611A/S612A/R860W displayed a ~2.5-fold (~2-fold under saturating conditions; see STAR Methods) reduced ATPase rate (Figure 3A). In contrast, ALC1^{fl} R860W and ALC1^{fl} R611A/S612A/R860W exhibited essentially identical DNA-stimulated ATPase activity (Figure S3A). The ALC1^{RLS} mutation also increased the Michaelis constant by a factor of ~5.3 and decreased the maximum rate for remodeling by a factor of ~6.4 (Figure 3B). Taken together, our data indicate that ALC1^{RLS} interactions play a role in nucleosome binding and in the regulation of maximum ATPase and remodeling activities of ALC1.

The AP mutation should not have a dramatic additional effect on remodeling by ALC1 with a mutated ALC1^{RLS} if both types of perturbations involved the same interface. Indeed, ALC1^{fl} R611A/S612A/R860W remodeled APM/APM nucleosomes with only moderately (by ~3.2-fold) reduced rates when compared to WT/WT nucleosomes (Figure 3C). In contrast, a ~13.7-fold change was observed when comparing the remodeling of WT/WT versus APM/APM nucleosomes by ALC1^{fl} R860W or a ~8.2-fold change for the remodeling of WT/WT nucleosomes by ALC1^{fl} R860W versus ALC1^{fl} R611A/R612A/R860W (Figure 3C). These observations are consistent with the ALC1^{RLS} and the AP contributing to the same interface. The observed residual sensitivity of ALC1^{fl} R611A/S612A/R860W to AP mutations most likely indicates that the R611A/S612A mutation of the regulatory linker does not completely abrogate its interaction with the AP. We cannot formally rule out an involvement of additional AP residues beyond the ones that we mutated or a contribution of additional elements of ALC1 to AP recognition. Interestingly, mutation of the Arg anchor (R611Q) alone reduced the remodeling activity of ALC1 by ~8.7-fold, close to the effect of

CellPress
OPEN ACCESS

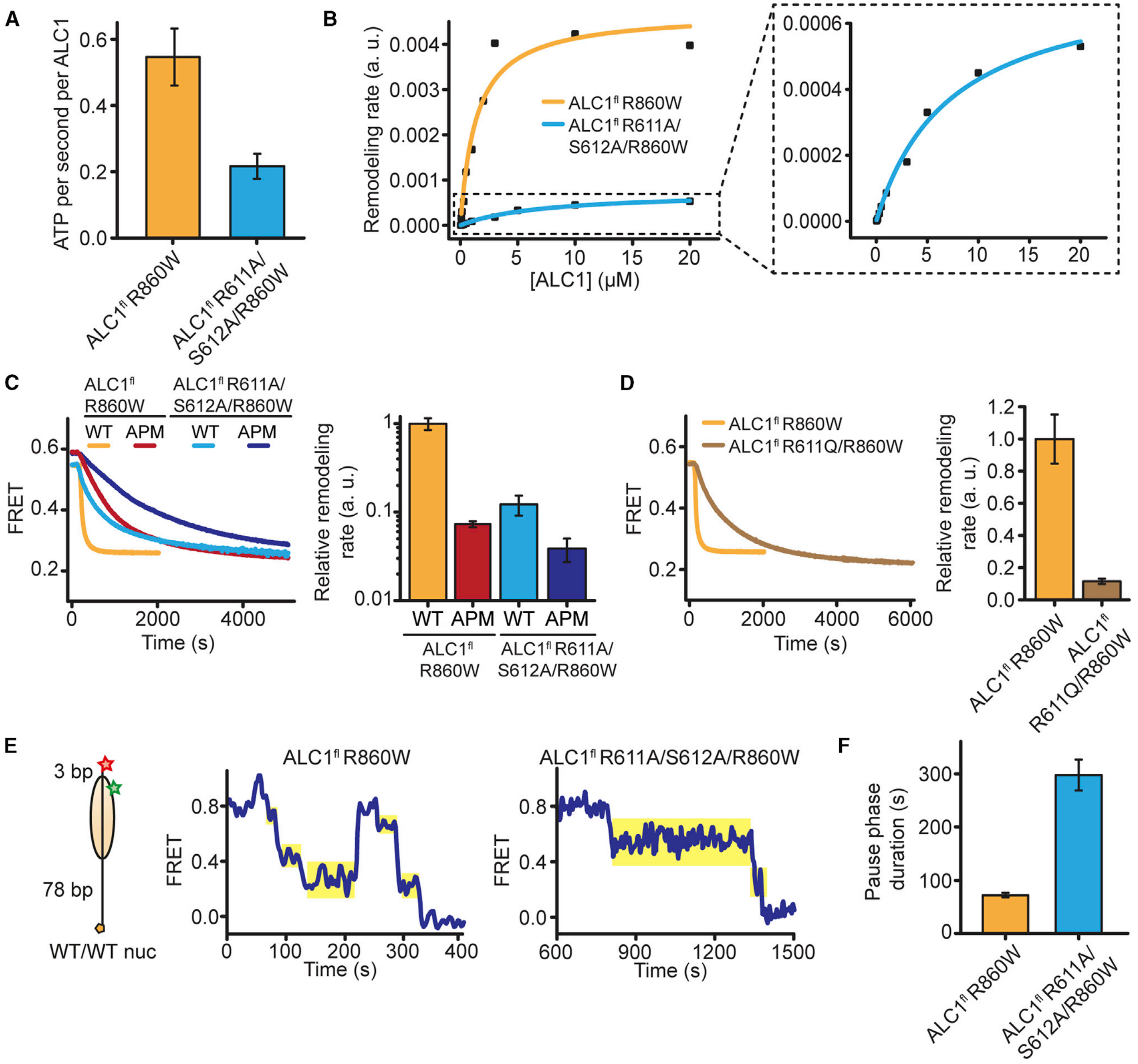

Figure 3. The Linker-AP Interaction Is Important for ALC1 Activity

(A) ATPase activity for 20 μM ALC1[fl] R860W or ALC1[fl] R611A/S612A/R860W in the presence of 2 μM WT/WT nucleosomes. Error bars represent SD (n = 3 independent experiments).

(B) Michaelis-Menten curves of remodeling rates for WT/WT nucleosomes (10 nM) and various ALC1[fl] R860W or R611A/S612A/R860W concentrations. Inset: enlarged view for ALC1[fl] R611A/S612A/R860W.

(C) Left: remodeling of 10 nM nucleosomes by 20 μM remodeler. Right: relative remodeling rates. Error bars represent SD (n = 3 independent experiments). Data for ALC1[fl] R860W and WT/WT nucleosomes as well as for ALC1[fl] R860W and APM/APM nucleosomes: same as indicated in Figure 1B.

(D) Left: remodeling of WT/WT nucleosomes (10 nM) in the presence of 20 μM ALC1[fl] R860W or ALC1[fl] R611Q. Right: relative rates for ALC1[fl] R860W or ALC1[fl] R611Q. Error bars represent SD (n = 3 independent experiments). Data for ALC1[fl] R860W and WT/WT nucleosomes: same as indicated in Figure 1B.

(E) Representative smFRET traces indicating the remodeling of individual WT/WT nucleosomes with 3 bp of linker DNA (left) by ALC1[fl] R860W (middle) or ALC1[fl] R611A/S612A/R860W (right). Only pauses immediately following translocation phases with a FRET decrease were analyzed (shaded yellow).

(F) Mean pause durations for ALC1[fl] R860W or ALC1[fl] R611A/S612A/R860W as described in (E). Error bars represent SEM (n > 170 events).

See also Figure S3.

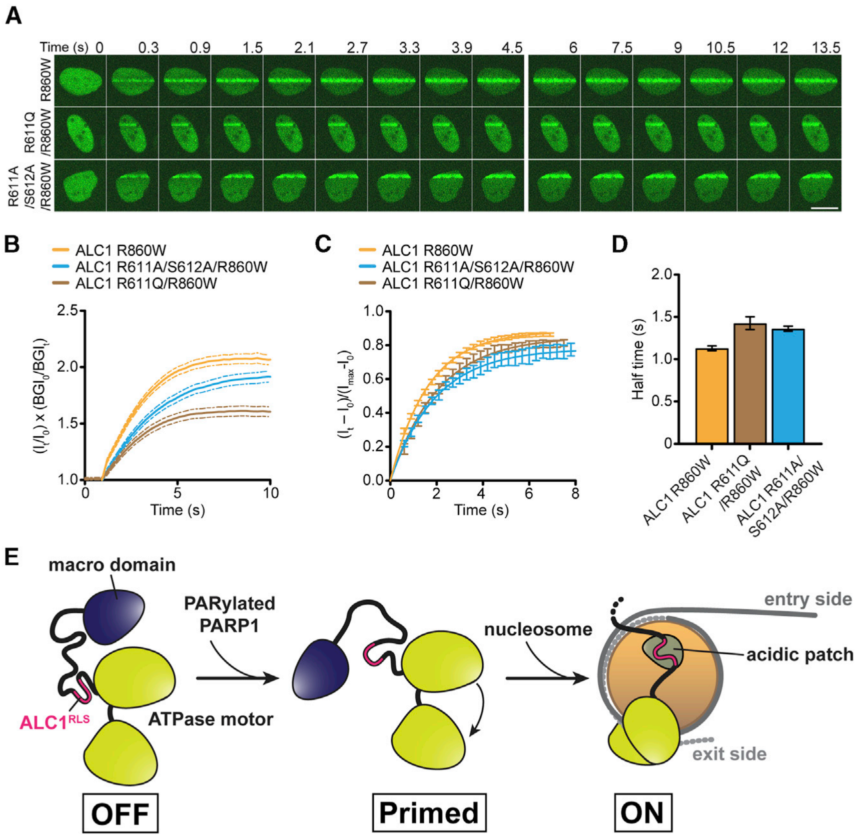

Figure 4. ALC1^{RLS} Mutations Alter Recruitment Dynamics

(A) U2OS cells expressing R860W, R611A/S612A/R860W, or R611Q/R860W YFP-ALC1, imaged upon laser damage. Scale bar, 10 μm.

(B) Kinetics of R860W, R611A/S612A/R860W, and R611Q/R860W YFP-ALC1 association with DNA breaks. Error bars represent SEM (n ≥ 131 traces from 3 independent experiments). See also Figure S4.

(C) Fractional recruitment of R860W, R611A/S612A/R860W, and R611Q/R860W YFP-ALC1 to DNA breaks. Error bars represent SEM (n ≥ 131 traces from 3 independent experiments).

(D) Half times from (C). Data are means ± 95% confidence intervals.

(E) In the absence of DNA damage, association of the macro domain of ALC1 with its C-terminal ATPase lobe stabilizes an ATPase "OFF" state. PAR binding to the macro domain displaces it from the ATPase. ALC1^{RLS} then tethers the remodeler to the AP, which stabilizes an active conformation (macro domain not shown) and promotes efficient coupling of ATP hydrolysis to nucleosome mobilization.

See also Figure S4.

the double-linker mutation (R611A/S612A) (Figure 3D). Consistent with our structure, the Arg anchor, therefore, plays a particularly important role in the linker-mediated regulation of ALC1.

To probe which phase of the remodeling cycle is affected by disrupting the ALC1^{RLS}-AP interaction, we monitored the remodeling of individual nucleosomes featuring only 3 bp of linker DNA on the acceptor-labeled side (Figure 3E). As previously observed for other remodelers (Blosser et al., 2009; Deindl et al., 2013; Gamarra et al., 2018; Hwang et al., 2014; Qiu et al., 2017), time traces exhibited phases of FRET change as well as translocation pauses. Mutation of the regulatory linker segment increased the average pause duration by over 4-fold (Figures 3E, 3F, and S3B–S3D), suggesting that interaction with the AP is important for exiting a paused state.

To probe the impact of the ALC1^{RLS} mutation on ALC1 function *in vivo*, we monitored recruitment kinetics at sites of DNA damage (Ahel et al., 2009) (Figures 4A–4D). Interestingly, both R611A/S612A and R611Q mutations notably compromised the overall extent of ALC1 recruitment (Figures 4A and 4B) but only modestly delayed recruitment kinetics (Figures 4C and 4D).

DISCUSSION

We have previously described the auto-inhibited conformation of ALC1, but how it is activated upon nucleosome binding remained unclear. In this work, we have discovered a regulatory motif within the ALC1 linker that promotes activation upon recognition of an intact AP.

An important role for the AP was suggested by ensemble experiments, where the mutation of both APs or the addition of LANA peptide compromised remodeling by ALC1. Our smFRET experiments revealed its overall ability to center nucleosomes, which was lost in the absence of an entry-side WT AP. Our cryo-EM structure of a nucleosome-ALC1 linker complex shows that the ALC1^{RLS} engages the AP via a conserved Arg anchor in a binding mode that closely resembles that of LANA, albeit with reverse N- to C-terminal polarity. Importantly, the ALC1^{RLS} mutation affected both the remodeling and ATPase rates under saturating conditions. Although the effect was modest (∼2-fold), the R611A/S612A linker mutation did not compromise ATPase activity indiscriminately but rather in a manner specific to nucleosomes. ALC1^{RLS} thus appears to contribute to a modest extent to the activation of the ALC1 ATPase, although our data do not explain how ALC1^{RLS}-AP interactions may promote an active ATPase conformation. Consistent with previous observations (Ahel et al., 2009; Gottschalk et al., 2009), the nucleosome-stimulated ATPase activity of ALC1^{fl} R860W was ∼1.6-fold higher than its DNA-stimulated ATPase activity. Mutation of ALC1^{RLS} affected the remodeling rate of ALC1 more strongly than its ATPase activity (∼6.4-fold versus ∼2-fold, respectively), suggesting a role of the ALC1^{RLS}-AP interaction in the coupling of ATP hydrolysis to nucleosome mobilization. Live-cell imaging showed that disrupting the ALC1-AP interaction impacts ALC1 recruitment to sites of DNA damage *in vivo*.

Our results demonstrate a critical role for the interaction between ALC1^{RLS} and the AP in tethering the remodeler to the

nucleosome and in stimulating both remodeling and, to a modest extent, the ATPase activities of ALC1. Taken together, our data are consistent with the following model for activation (Figure 4E): upon recruitment to DNA damage and displacement of the macro domain, ALC1 associates with the nucleosome (Lehmann et al., 2017). In the fully activated state of ALC1, the AP engages the ALC1^{RLS}, which tethers the remodeler to the nucleosome and promotes efficient coupling of ATP hydrolysis to remodeling.

Our work has uncovered an unexpected function of the ALC1^{RLS} that enables full activation only upon specific recognition of a nucleosome with an intact AP. Our experiments reveal that, with both APs intact, ALC1 exhibits a preference to center the octamer, albeit to a lesser extent than ISWI remodelers such as ACF and ISW2 (Ito et al., 1997; Längst et al., 1999; Tsukiyama et al., 1999; Varga-Weisz et al., 1997). Our data further reveal an unexpected divergence between ALC1 and the related Chd1 in their response to AP asymmetry. Unlike ALC1, Chd1 is only modestly affected by AP mutations (Levendosky and Bowman, 2019). Moreover, Chd1 responds similarly to AP mutations on entry-side and exit-side H2A/H2B dimers. In stark contrast, ALC1 depends much more strongly on a WT AP on the entry side. For ALC1-catalyzed remodeling of nucleosomes with AP asymmetry, net movement occurs toward the WT AP-containing side. Our smFRET analyses suggest that the pause phase of the remodeling cycle might serve to probe for an intact AP. Although future studies with histone variants are required, we speculate that, *in vivo*, the selective removal or blocking of one of the two APs might locally redirect ALC1 remodeling away from spacing and toward the disorganization of nucleosomes. Such remodeling may facilitate chromatin relaxation in a DNA-damage-specific chromatin context.

STAR★METHODS

Detailed methods are provided in the online version of this paper and include the following:

- KEY RESOURCES TABLE
- RESOURCE AVAILABILITY
  - Lead Contact
  - Materials Availability
  - Data and Code Availability
- EXPERIMENTAL MODEL AND SUBJECT DETAILS
- METHOD DETAILS
  - Cloning, plasmids, and cell lines
  - DNA constructs
  - Peptides
  - ALC1 expression and purification
  - PARP1 expression and purification
  - Histone expression and purification
  - Histone labeling
  - Histone octamer assembly
  - Nucleosome assembly
  - ATPase activity measurements
  - Fluorescence anisotropy binding assay
  - Ensemble FRET assay for nucleosome remodeling
  - Single-molecule FRET remodeling
  - Cross-linking coupled to mass spectrometry
  - Cryo-EM sample preparation
  - Cryo-EM data collection and processing
  - Model building and refinement
  - Whole-cell extracts and immunoblotting
  - Laser damage
- QUANTIFICATION AND STATISTICAL ANALYSIS

SUPPLEMENTAL INFORMATION

Supplemental Information can be found online at https://doi.org/10.1016/j.celrep.2020.108529.

ACKNOWLEDGMENTS

We thank S.D. Knight for insightful comments; M. Hällberg, M. Carroni, and J. Conrad for assistance with cryo-EM data collection; J. Locke for early cryo-EM work; and A. Emmerich for preliminary smFRET experiments. This work was supported by the European Research Council (StG, 714068 to S.D.; AdG, TelMetab to S.J.B.; and CoG, 820102 to A.C.), the KAW Foundation (grant KAW 019.0306), the Swedish Research Council (VR grant 2019-03534), and Cancerfonden (grant 19 0055 Pj). Work at the Francis Crick Institute was funded by CRUK, Wellcome Trust, and MRC (FC001065 to A.C. and FC0010048 to S.J.B.). S.J.B. acknowledges support from Wellcome Trust Senior Investigator and collaborative grants, and S.T. acknowledges support from NIH NIGMS grant R35 GM127034. S.D. is an EMBO Young Investigator.

AUTHOR CONTRIBUTIONS

S.D. conceived and oversaw the study. L.C.L., L.B., G.H., K.B., A.S., G.G., S.P., G.D., H.O., S.T., A.C., J.M.S., S.J.B., and S.D. designed and/or conducted experiments. A.S. collected/analyzed smFRET data. L.B. collected cryo-EM data, with help from G.G. G.G. analyzed cryo-EM data, with help from L.B. S.D. and S.J.B. wrote the manuscript, with input from all authors.

DECLARATION OF INTERESTS

The authors declare no competing interests.

Received: July 10, 2020
Revised: September 8, 2020
Accepted: November 24, 2020
Published: December 22, 2020

REFERENCES

Ahel, D., Horejsí, Z., Wiechens, N., Polo, S.E., Garcia-Wilson, E., Ahel, I., Flynn, H., Skehel, M., West, S.C., Jackson, S.P., et al. (2009). Poly(ADP-ribose)-dependent regulation of DNA repair by the chromatin remodeling enzyme ALC1. Science *325*, 1240–1243.

Ayala, R., Willhoft, O., Aramayo, R.J., Wilkinson, M., McCormack, E.A., Ocloo, L., Wigley, D.B., and Zhang, X. (2018). Structure and regulation of the human INO80-nucleosome complex. Nature *556*, 391–395.

Barbera, A.J., Chodaparambil, J.V., Kelley-Clarke, B., Joukov, V., Walter, J.C., Luger, K., and Kaye, K.M. (2006). The nucleosomal surface as a docking station for Kaposi's sarcoma herpesvirus LANA. Science *311*, 856–861.

Bartholomew, B. (2014). Regulating the chromatin landscape: structural and mechanistic perspectives. Annu. Rev. Biochem. *83*, 671–696.

Becker, P.B., and Workman, J.L. (2013). Nucleosome remodeling and epigenetics. Cold Spring Harb. Perspect. Biol. *5*, a017905.

Bilokapic, S., Strauss, M., and Halic, M. (2018). Histone octamer rearranges to adapt to DNA unwrapping. Nat. Struct. Mol. Biol. *25*, 101–108.

Blosser, T.R., Yang, J.G., Stone, M.D., Narlikar, G.J., and Zhuang, X. (2009). Dynamics of nucleosome remodelling by individual ACF complexes. Nature *462*, 1022–1027.

Bowman, G.D. (2010). Mechanisms of ATP-dependent nucleosome sliding. Curr. Opin. Struct. Biol. *20*, 73–81.

Casañal, A., Lohkamp, B., and Emsley, P. (2020). Current developments in Coot for macromolecular model building of Electron Cryo-microscopy and Crystallographic Data. Protein Sci. *29*, 1069–1078.

Chen, M., Huang, J.-D., Hu, L., Zheng, B.-J., Chen, L., Tsang, S.L., and Guan, X.-Y. (2009). Transgenic CHD1L expression in mouse induces spontaneous tumors. PLoS ONE *4*, e6727.

Chen, L., Chan, T.H.M., Yuan, Y.-F., Hu, L., Huang, J., Ma, S., Wang, J., Dong, S.-S., Tang, K.H., Xie, D., et al. (2010). CHD1L promotes hepatocellular carcinoma progression and metastasis in mice and is associated with these processes in human patients. J. Clin. Invest. *120*, 1178–1191.

Clapier, C.R., Iwasa, J., Cairns, B.R., and Peterson, C.L. (2017). Mechanisms of action and regulation of ATP-dependent chromatin-remodelling complexes. Nat. Rev. Mol. Cell Biol. *18*, 407–422.

Croll, T.I. (2018). ISOLDE: a physically realistic environment for model building into low-resolution electron-density maps. Acta Crystallogr. D Struct. Biol. *74*, 519–530.

D'Amours, D., Desnoyers, S., D'Silva, I., and Poirier, G.G. (1999). Poly(ADP-ribosyl)ation reactions in the regulation of nuclear functions. Biochem. J. *342*, 249–268.

Dann, G.P., Liszczak, G.P., Bagert, J.D., Müller, M.M., Nguyen, U.T.T., Wojcik, F., Brown, Z.Z., Bos, J., Panchenko, T., Pihl, R., et al. (2017). ISWI chromatin remodellers sense nucleosome modifications to determine substrate preference. Nature *548*, 607–611.

Dao, H.T., Dul, B.E., Dann, G.P., Liszczak, G.P., and Muir, T.W. (2020). A basic motif anchoring ISWI to nucleosome acidic patch regulates nucleosome spacing. Nat. Chem. Biol. *16*, 134–142.

de Murcia, G., Huletsky, A., Lamarre, D., Gaudreau, A., Pouyet, J., Daune, M., and Poirier, G.G. (1986). Modulation of chromatin superstructure induced by poly(ADP-ribose) synthesis and degradation. J. Biol. Chem. *261*, 7011–7017.

Deindl, S., and Zhuang, X. (2012). Monitoring conformational dynamics with single-molecule fluorescence energy transfer: applications in nucleosome remodeling. Methods Enzymol. *513*, 59–86.

Deindl, S., Hwang, W.L., Hota, S.K., Blosser, T.R., Prasad, P., Bartholomew, B., and Zhuang, X. (2013). ISWI remodelers slide nucleosomes with coordinated multi-base-pair entry steps and single-base-pair exit steps. Cell *152*, 442–452.

Dyer, P.N., Edayathumangalam, R.S., White, C.L., Bao, Y., Chakravarthy, S., Muthurajan, U.M., and Luger, K. (2004). Reconstitution of nucleosome core particles from recombinant histones and DNA. Methods Enzymol. *375*, 23–44.

Edelstein, A., Amodaj, N., Hoover, K., Vale, R., and Stuurman, N. (2010). Computer control of microscopes using μManager. Curr. Protoc. Mol. Biol., Chapter 14, Unit 14.20.

El-Khamisy, S.F., Masutani, M., Suzuki, H., and Caldecott, K.W. (2003). A requirement for PARP-1 for the assembly or stability of XRCC1 nuclear foci at sites of oxidative DNA damage. Nucleic Acids Res. *31*, 5526–5533.

England, J.R., Huang, J., Jennings, M.J., Makde, R.D., and Tan, S. (2010). RCC1 uses a conformationally diverse loop region to interact with the nucleosome: a model for the RCC1-nucleosome complex. J. Mol. Biol. *398*, 518–529.

Eustermann, S., Schall, K., Kostrewa, D., Lakomek, K., Strauss, M., Moldt, M., and Hopfner, K.-P. (2018). Structural basis for ATP-dependent chromatin remodelling by the INO80 complex. Nature *556*, 386–390.

Flaus, A., Martin, D.M.A., Barton, G.J., and Owen-Hughes, T. (2006). Identification of multiple distinct Snf2 subfamilies with conserved structural motifs. Nucleic Acids Res. *34*, 2887–2905.

Frechette, A., Huletsky, A., Aubin, R.J., de Murcia, G., Mandel, P., Lord, A., Grondin, G., and Poirier, G.G. (1985). Poly(ADP-ribosyl)ation of chromatin: kinetics of relaxation and its effect on chromatin solubility. Can. J. Biochem. Cell Biol. *63*, 764–773.

Gamarra, N., Johnson, S.L., Trnka, M.J., Burlingame, A.L., and Narlikar, G.J. (2018). The nucleosomal AP relieves auto-inhibition by the ISWI remodeler SNF2h. eLife *7*, e35322.

Goddard, T.D., Huang, C.C., Meng, E.C., Pettersen, E.F., Couch, G.S., Morris, J.H., and Ferrin, T.E. (2018). UCSF ChimeraX: Meeting modern challenges in visualization and analysis. Protein Sci. *27*, 14–25.

Goldman, J.A., Garlick, J.D., and Kingston, R.E. (2010). Chromatin remodeling by imitation switch (ISWI) class ATP-dependent remodelers is stimulated by histone variant H2A.Z. J. Biol. Chem. *285*, 4645–4651.

Gottschalk, A.J., Timinszky, G., Kong, S.E., Jin, J., Cai, Y., Swanson, S.K., Washburn, M.P., Florens, L., Ladurner, A.G., Conaway, J.W., and Conaway, R.C. (2009). Poly(ADP-ribosyl)ation directs recruitment and activation of an ATP-dependent chromatin remodeler. Proc. Natl. Acad. Sci. USA *106*, 13770–13774.

Gottschalk, A.J., Trivedi, R.D., Conaway, J.W., and Conaway, R.C. (2012). Activation of the SNF2 family ATPase ALC1 by poly(ADP-ribose) in a stable ALC1·PARP1·nucleosome intermediate. J. Biol. Chem. *287*, 43527–43532.

Hassa, P.O., Haenni, S.S., Elser, M., and Hottiger, M.O. (2006). Nuclear ADP-ribosylation reactions in mammalian cells: where are we today and where are we going? Microbiol. Mol. Biol. Rev. *70*, 789–829.

He, S., Wu, Z., Tian, Y., Yu, Z., Yu, J., Wang, X., Li, J., Liu, B., and Xu, Y. (2020). Structure of nucleosome-bound human BAF complex. Science *367*, 875–881.

Hwang, W.L., Deindl, S., Harada, B.T., and Zhuang, X. (2014). Histone H4 tail mediates allosteric regulation of nucleosome remodelling by linker DNA. Nature *512*, 213–217.

Ito, T., Bulger, M., Pazin, M.J., Kobayashi, R., and Kadonaga, J.T. (1997). ACF, an ISWI-containing and ATP-utilizing chromatin assembly and remodeling factor. Cell *90*, 145–155.

Klinker, H., Haas, C., Harrer, N., Becker, P.B., and Mueller-Planitz, F. (2014). Rapid purification of recombinant histones. PLoS ONE *9*, e104029.

Langelier, M.-F., Steffen, J.D., Riccio, A.A., McCauley, M., and Pascal, J.M. (2017). Purification of DNA Damage-Dependent PARPs from E. coli for Structural and Biochemical Analysis. Methods Mol. Biol. *1608*, 431–444.

Längst, G., Bonte, E.J., Corona, D.F.V., and Becker, P.B. (1999). Nucleosome movement by CHRAC and ISWI without disruption or trans-displacement of the histone octamer. Cell *97*, 843–852.

Leduc, Y., de Murcia, G., Lamarre, D., and Poirier, G.G. (1986). Visualization of poly(ADP-ribose) synthetase associated with polynucleosomes by immunoelectron microscopy. Biochim. Biophys. Acta *885*, 248–255.

Lehmann, L.C., Hewitt, G., Aibara, S., Leitner, A., Marklund, E., Maslen, S.L., Maturi, V., Chen, Y., van der Spoel, D., Skehel, J.M., et al. (2017). Mechanistic Insights into Autoinhibition of the Oncogenic Chromatin Remodeler ALC1. Mol. Cell *68*, 847–859.e7.

Levendosky, R.F., and Bowman, G.D. (2019). Asymmetry between the two acidic patches dictates the direction of nucleosome sliding by the ISWI chromatin remodeler. eLife *8*, e45472.

Levendosky, R.F., Sabantsev, A., Deindl, S., and Bowman, G.D. (2016). The Chd1 chromatin remodeler shifts hexasomes unidirectionally. eLife *5*, e21356.

Liebschner, D., Afonine, P.V., Baker, M.L., Bunkóczi, G., Chen, V.B., Croll, T.I., Hintze, B., Hung, L.-W., Jain, S., McCoy, A.J., et al. (2019). Macromolecular structure determination using X-rays, neutrons and electrons: recent developments in Phenix. Acta Crystallogr. D Struct. Biol. *75*, 861–877.

Lindahl, T., Satoh, M.S., and Dianov, G. (1995). Enzymes acting at strand interruptions in DNA. Philos. Trans. R. Soc. Lond. B Biol. Sci. *347*, 57–62.

Lorch, Y., and Kornberg, R.D. (2017). Chromatin-remodeling for transcription. Q. Rev. Biophys. *50*, e5.

Lowary, P.T., and Widom, J. (1998). New DNA sequence rules for high affinity binding to histone octamer and sequence-directed nucleosome positioning. J. Mol. Biol. *276*, 19–42.

Luger, K., Mäder, A.W., Richmond, R.K., Sargent, D.F., and Richmond, T.J. (1997). Crystal structure of the nucleosome core particle at 2.8 A resolution. Nature *389*, 251–260.

Lusser, A., and Kadonaga, J.T. (2003). Chromatin remodeling by ATP-dependent molecular machines. BioEssays *25*, 1192–1200.

CellPress
OPEN ACCESS

Ma, N.-F., Hu, L., Fung, J.M., Xie, D., Zheng, B.-J., Chen, L., Tang, D.-J., Fu, L., Wu, Z., Chen, M., et al. (2008). Isolation and characterization of a novel oncogene, amplified in liver cancer 1, within a commonly amplified region at 1q21 in hepatocellular carcinoma. Hepatology *47*, 503–510.

Malanga, M., and Althaus, F.R. (2005). The role of poly(ADP-ribose) in the DNA damage signaling network. Biochem. Cell Biol. *83*, 354–364.

Marchio, A., Meddeb, M., Pineau, P., Danglot, G., Tiollais, P., Bernheim, A., and Dejean, A. (1997). Recurrent chromosomal abnormalities in hepatocellular carcinoma detected by comparative genomic hybridization. Genes Chromosomes Cancer *18*, 59–65.

McGinty, R.K., and Tan, S. (2016). Recognition of the nucleosome by chromatin factors and enzymes. Curr. Opin. Struct. Biol. *37*, 54–61.

Mu, Q.-J., Li, H.-L., Yao, Y., Liu, S.-C., Yin, C.-G., and Ma, X.-Z. (2015). Chromodomain Helicase/ATPase DNA-Binding Protein 1-Like Gene (CHD1L) Expression and Implications for Invasion and Metastasis of Breast Cancer. PLoS ONE *10*, e0143030.

Narlikar, G.J., Sundaramoorthy, R., and Owen-Hughes, T. (2013). Mechanisms and functions of ATP-dependent chromatin-remodeling enzymes. Cell *154*, 490–503.

Ngo, T.T.M., Zhang, Q., Zhou, R., Yodh, J.G., and Ha, T. (2015). Asymmetric unwrapping of nucleosomes under tension directed by DNA local flexibility. Cell *160*, 1135–1144.

Pines, A., Vrouwe, M.G., Marteijn, J.A., Typas, D., Luijsterburg, M.S., Cansoy, M., Hensbergen, P., Deelder, A., de Groot, A., Matsumoto, S., et al. (2012). PARP1 promotes nucleotide excision repair through DDB2 stabilization and recruitment of ALC1. J. Cell Biol. *199*, 235–249.

Poirier, G.G., de Murcia, G., Jongstra-Bilen, J., Niedergang, C., and Mandel, P. (1982). Poly(ADP-ribosyl)ation of polynucleosomes causes relaxation of chromatin structure. Proc. Natl. Acad. Sci. USA *79*, 3423–3427.

Punjani, A., Rubinstein, J.L., Fleet, D.J., and Brubaker, M.A. (2017). cryoSPARC: algorithms for rapid unsupervised cryo-EM structure determination. Nat. Methods *14*, 290–296.

Qiu, Y., Levendosky, R.F., Chakravarthy, S., Patel, A., Bowman, G.D., and Myong, S. (2017). The Chd1 chromatin remodeler shifts nucleosomal DNA bidirectionally as a monomer. Mol. Cell *68*, 76–88.e6.

Ran, F.A., Hsu, P.D., Wright, J., Agarwala, V., Scott, D.A., and Zhang, F. (2013). Genome engineering using the CRISPR-Cas9 system. Nat. Protoc. *8*, 2281–2308.

Rasnik, I., McKinney, S.A., and Ha, T. (2006). Nonblinking and long-lasting single-molecule fluorescence imaging. Nat. Methods *3*, 891–893.

Realini, C.A., and Althaus, F.R. (1992). Histone shuttling by poly(ADP-ribosylation). J. Biol. Chem. *267*, 18858–18865.

Sabantsev, A., Levendosky, R.F., Zhuang, X., Bowman, G.D., and Deindl, S. (2019). Direct observation of coordinated DNA movements on the nucleosome during chromatin remodelling. Nat. Commun. *10*, 1720.

Satoh, M.S., and Lindahl, T. (1992). Role of poly(ADP-ribose) formation in DNA repair. Nature *356*, 356–358.

Scheres, S.H.W. (2012). RELION: implementation of a Bayesian approach to cryo-EM structure determination. J. Struct. Biol. *180*, 519–530.

Schindelin, J., Arganda-Carreras, I., Frise, E., Kaynig, V., Longair, M., Pietzsch, T., Preibisch, S., Rueden, C., Saalfeld, S., Schmid, B., et al. (2012). Fiji: an open-source platform for biological-image analysis. Nat. Methods *9*, 676–682.

Schneider, C.A., Rasband, W.S., and Eliceiri, K.W. (2012). NIH Image to ImageJ: 25 years of image analysis. Nat. Methods *9*, 671–675.

Sellou, H., Lebeaupin, T., Chapuis, C., Smith, R., Hegele, A., Singh, H.R., Kozlowski, M., Bultmann, S., Ladurner, A.G., Timinszky, G., and Huet, S. (2016). The poly(ADP-ribose)-dependent chromatin remodeler Alc1 induces local chromatin relaxation upon DNA damage. Mol. Biol. Cell *27*, 3791–3799.

Singh, H.R., Nardozza, A.P., Möller, I.R., Knobloch, G., Kistemaker, H.A.V., Hassler, M., Harrer, N., Blessing, C., Eustermann, S., Kotthoff, C., et al. (2017). A Poly-ADP-Ribose Trigger Releases the Auto-Inhibition of a Chromatin Remodeling Oncogene. Mol. Cell *68*, 860–871.e7.

Smith, R., Sellou, H., Chapuis, C., Huet, S., and Timinszky, G. (2018). CHD3 and CHD4 recruitment and chromatin remodeling activity at DNA breaks is promoted by early poly(ADP-ribose)-dependent chromatin relaxation. Nucleic Acids Res. *46*, 6087–6098.

Smith, R., Lebeaupin, T., Juhász, S., Chapuis, C., D'Augustin, O., Dutertre, S., Burkovics, P., Biertümpfel, C., Timinszky, G., and Huet, S. (2019). Poly(ADP-ribose)-dependent chromatin unfolding facilitates the association of DNA-binding proteins with DNA at sites of damage. Nucleic Acids Res. *47*, 11250–11267.

Tegunov, D., and Cramer, P. (2019). Real-time cryo-electron microscopy data preprocessing with Warp. Nat. Methods *16*, 1146–1152.

Tsukiyama, T., Palmer, J., Landel, C.C., Shiloach, J., and Wu, C. (1999). Characterization of the imitation switch subfamily of ATP-dependent chromatin-remodeling factors in Saccharomyces cerevisiae. Genes Dev. *13*, 686–697.

Valencia, A.M., Collings, C.K., Dao, H.T., St Pierre, R., Cheng, Y.-C., Huang, J., Sun, Z.-Y., Seo, H.-S., Mashtalir, N., Comstock, D.E., et al. (2019). Recurrent SMARCB1 Mutations Reveal a Nucleosome Acidic Patch Interaction Site That Potentiates mSWI/SNF Complex Chromatin Remodeling. Cell *179*, 1342–1356.e23.

Varga-Weisz, P.D., Wilm, M., Bonte, E., Dumas, K., Mann, M., and Becker, P.B. (1997). Chromatin-remodelling factor CHRAC contains the ATPases ISWI and topoisomerase II. Nature *388*, 598–602.

Vasudevan, D., Chua, E.Y.D., and Davey, C.A. (2010). Crystal structures of nucleosome core particles containing the '601' strong positioning sequence. J. Mol. Biol. *403*, 1–10.

Wagner, F.R., Dienemann, C., Wang, H., Stützer, A., Tegunov, D., Urlaub, H., and Cramer, P. (2020). Structure of SWI/SNF chromatin remodeller RSC bound to a nucleosome. Nature *579*, 448–451.

Willhoft, O., Bythell-Douglas, R., McCormack, E.A., and Wigley, D.B. (2016). Synergy and antagonism in regulation of recombinant human INO80 chromatin remodeling complex. Nucleic Acids Res. *44*, 8179–8188.

Willhoft, O., Ghoneim, M., Lin, C.-L., Chua, E.Y.D., Wilkinson, M., Chaban, Y., Ayala, R., McCormack, E.A., Ocloo, L., Rueda, D.S., and Wigley, D.B. (2018). Structure and dynamics of the yeast SWR1-nucleosome complex. Science *362*, eaat7716.

Wong, N., Chan, A., Lee, S.-W., Lam, E., To, K.-F., Lai, P.B.-S., Li, X.-N., Liew, C.-T., and Johnson, P.J. (2003). Positional mapping for amplified DNA sequences on 1q21-q22 in hepatocellular carcinoma indicates candidate genes over-expression. J. Hepatol. *38*, 298–306.

Yang, J.G., Madrid, T.S., Sevastopoulos, E., and Narlikar, G.J. (2006). The chromatin-remodeling enzyme ACF is an ATP-dependent DNA length sensor that regulates nucleosome spacing. Nat. Struct. Mol. Biol. *13*, 1078–1083.

Ye, Y., Wu, H., Chen, K., Clapier, C.R., Verma, N., Zhang, W., Deng, H., Cairns, B.R., Gao, N., and Chen, Z. (2019). Structure of the RSC complex bound to the nucleosome. Science *366*, 838–843.

Zhang, K. (2016). Gctf: Real-time CTF determination and correction. J. Struct. Biol. *193*, 1–12.

Zheng, S.Q., Palovcak, E., Armache, J.-P., Verba, K.A., Cheng, Y., and Agard, D.A. (2017). MotionCor2: anisotropic correction of beam-induced motion for improved cryo-electron microscopy. Nat. Methods *14*, 331–332.

Zhou, K., Gaullier, G., and Luger, K. (2019). Nucleosome structure and dynamics are coming of age. Nat. Struct. Mol. Biol. *26*, 3–13.

Zivanov, J., Nakane, T., Forsberg, B.O., Kimanius, D., Hagen, W.J., Lindahl, E., and Scheres, S.H. (2018). New tools for automated high-resolution cryo-EM structure determination in RELION-3. eLife *7*, e42166.

Zivanov, J., Nakane, T., and Scheres, S.H.W. (2020). Estimation of high-order aberrations and anisotropic magnification from cryo-EM data sets in *RELION*-3.1. IUCrJ *7*, 253–267.

STAR★METHODS

KEY RESOURCES TABLE

REAGENT or RESOURCE	SOURCE	IDENTIFIER
Antibodies		
hnRNPA1	Sant Cruz Biotechnology	Cat#SC-32301; RRID: AB_627729
CHD1L	Cell Signaling Technology	Cat#13460; RRID: AB_2798225
Bacterial and Virus Strains		
E. coli Rosetta 2 (DE3)	Novagen	Cat#71400
E. coli BL21 (DE3) pLysS	Novagen	Cat#69451
Chemicals, Peptides, and Recombinant Proteins		
BrdU	Sigma-Aldrich	Cat#B5002
Doxycycline	Sigma-Aldrich	Cat#M0503-5X2MG
Blasticidin	ThemoFisher Scientific	Cat#A1113903
Hygromycin B	ThemoFisher Scientific	Cat#10687010
Zeocin	ThemoFisher Scientific	Cat#R25005
Lipofectamine 2000	ThemoFisher Scientific	Cat#11668019
EDTA-free Complete protease inhibitor cocktail	Roche	Cat#COEDTAF-RO
PhosSTOP phosphatase inhibitor cocktail	Roche	Cat#PHOSS-RO
4x NuPAGE LDS sample buffer	ThemoFisher Scientific	Cat#NP0008
LANA peptide	Peptide 2.0	N/A
LANA peptide-TAMRA	Peptide 2.0	N/A
ALC1^{RLS} peptide	Peptide 2.0	N/A
ALC1^{RLS} peptide-TAMRA	Peptide 2.0	N/A
ALC1 linker peptide-Biotin	Peptide 2.0	N/A
Protease inhibitor cocktail	Sigma-Aldrich	Cat#5056489001
Benzonase Nuclease	Sigma-Aldrich	Cat#E1014
Cy3-maleimide	GE Healthcare	Cat#PA23031
Pyruvate Kinase/Lactic Dehydrogenase	Sigma-Aldrich	Cat#P0294
Trypsin	Promega UK	Cat#V5111
Glu-C	Promega UK	Cat#V165A
Deposited Data		
Raw cryo-EM videos and final particle images (deposited on EMPIAR)	This study	EMPIAR-10465
Nucleosome cryo-EM maps (deposited on EMDB)	This study	EMD-11220
Hexasome cryo-EM maps (deposited on EMDB)	This study	EMD-11221
Nucleosome atomic model (deposited in PDB)	This study	6ZHX
Hexasome atomic model (deposited in PDB)	This study	6ZHY
Experimental Models: Cell Lines		
U2OS Flp-In T-Rex HOST	Durocher lab	N/A
ALC1 –/– U2OS Flp-In T-REx	This paper	N/A
ALC1 –/– U2OS Flp-In YFP-ALC1	This paper	N/A
ALC1 –/– U2OS Flp-In YFP-ALC1 R860W	This paper	N/A
ALC1 –/– U2OS Flp-In YFP-ALC1 R611A/ R612AR860W	This paper	N/A
ALC1 –/– U2OS Flp-In YFP-ALC1 R611Q R860W	This paper	N/A
Oligonucleotides		
DNA oligonucleotides	This paper	Table S3

(*Continued on next page*)

Continued

REAGENT or RESOURCE	SOURCE	IDENTIFIER
Recombinant DNA		
Human ALC1[fl] (16-879) pNIC-CH2	Lehmann et al., 2017	N/A
Human PARP1 (1-1014) pET-28	Langelier et al., 2017	N/A
px459	Addgene	#62988
px459 ALC1 EX3	This paper	N/A
pOG44 Flp-Recombinase expression vector	ThemoFisher Scientific	Cat#V600520
ALC1 WT YFP-ALC1 pDEST-YFP/FRT/TO	Ahel et al., 2009	N/A
ALC1 R860W YFP-ALC1 pDEST-YFP/FRT/TO	Lehmann et al., 2017	N/A
ALC1 R611A/R612AR860W YFP-ALC1 pDEST-YFP/FRT/TO	This paper	N/A
ALC1 R611Q/R860W YFP-ALC1 pDEST-YFP/FRT/TO	This paper	N/A
X. laevis Pet3a_H2A	Geeta Narlikar Lab	N/A
X. laevis Pet3a_H2B	Geeta Narlikar Lab	N/A
X. laevis Pet3a_H3	Geeta Narlikar Lab	N/A
X. laevis Pet3a_H4	Geeta Narlikar Lab	N/A
Software and Algorithms		
Warp	Tegunov and Cramer, 2019	http://www.warpem.com/warp/#
cryoSPARC	Punjani et al., 2017	https://cryosparc.com/
RELION-3.1	Scheres, 2012; Zivanov et al., 2018, 2020	https://www3.mrc-lmb.cam.ac.uk/relion/index.php/Main_Page
UCSF ChimeraX	Goddard et al., 2018	https://www.rbvi.ucsf.edu/chimerax/
Coot	Casañal et al., 2020	https://www2.mrc-lmb.cam.ac.uk/personal/pemsley/coot/
ISOLDE	Croll, 2018	https://isolde.cimr.cam.ac.uk/
Prism 8	GraphPad Software	https://www.graphpad.com/
Fiji	NIH	https://imagej.net/Fiji/Downloads
Image Lab 5.2.1	Bio-Rad Laboratories	https://www.bio-rad.com/en-uk/product/image-lab-%20software?ID%20=%20KRE6P5E8Z
Andor iQ.	Oxford Instruments	https://andor.oxinst.com/products/iq-live-cell-imaging-software/
Adobe Illustrator 23.11	Adobe	https://www.adobe.com/uk/products/illustrator.html
Phenix suite	Liebschner et al., 2019	https://www.phenix-online.org/
Other		
5 mL HisTrap FF	GE Healthcare	Cat#17525501
HiPrep 26/10 Desalting	GE Healthcare	Cat#17508701
5 mL HiTrap Q HP	GE Healthcare	Cat#17115401
5 mL HiTrap SP HP	GE Healthcare	Cat#17115201
HiLoad 16/600 Superdex 200 pg	GE Healthcare	Cat#28989335
5 mL HiTrap Heparin HP	GE Healthcare	Cat#17040703
HiPrep 16/60 Sephacryl S-200 HR	GE Healthcare	Cat#17116601
Superdex Peptide 3.2/300 (discontinued)	GE Healthcare	Cat# 29036231
Acquity UPLC CSH C18 1.7 μm, 1.0 × 100 mm column	Waters	Cat#176002137
C18 Acclaim PepMap100 5 μm, 100 μm × 20 mm nanoViper	Thermo Scientific	Cat#164564
C18 Acclaim PepMap100 3 μm, 75 μm × 250 mm nanoViper	Thermo Scientific	Cat#164569
Pierce Monomeric Avidin Agarose resin	Thermo Scientific	Cat#20228
Quantifoil R 2/2 Cu 300 grids	Electron Microscopy Sciences	Cat#Q3100CR2

RESOURCE AVAILABILITY

Lead Contact

Further information and requests for resources and reagents should be directed to and will be fulfilled by the Lead Contact, Sebastian Deindl (sebastian.deindl@icm.uu.se).

Materials Availability

This study did not generate new unique reagents.

Data and Code Availability

Raw cryo-EM videos and final particle images: accession code EMPIAR-10465. EMDB accession codes: EMD-11220 (nucleosome) and EMD-11221 (hexasome). PDB accession codes: 6ZHX (nucleosome) and 6ZHY (hexasome).

EXPERIMENTAL MODEL AND SUBJECT DETAILS

cDNAs for ALC1 and PARP1 were of *Homo sapiens* origin. Histone proteins (recombinantly expressed in *E. coli*) were of *Xenopus laevis* origin. Cell lines as well as growth conditions are described in the Method Details.

METHOD DETAILS

Cloning, plasmids, and cell lines

Oligonucleotides used in this study were ordered from IDT (see Table S3). The ALC1^{fl} (16-879) construct contains the human ALC1 sequence and a 6xHis-tag, as previously published (Lehmann et al., 2017). All point mutations were generated by PCR-based site-directed mutagenesis. The plasmid for human PARP1 (1-1014) with an N-terminal 6xHis-tag was a kind gift from John M. Pascal (Langelier et al., 2017).

U2OS Flp-In T-REx cells were a kind gift from Durocher lab and maintained in DMEM medium (GIBCO/Thermo Fisher) supplemented with 10% FBS and Pen/Strep with 15.5 μg/ml zeocin (Invitrogen) and 4 μg/ml blasticidin. CRISPR inactivation of ALC1 was carried out in Flp-In T-REx U2OS cells by transiently expressing Cas9 and gRNA targeting Exon3 of ALC1 F-oligo: CACCGCAG GAAGATTAAATGATGAA R-oligo: AAACTTCATCATTTAATCTTCCTGC with px459 (addgene) (Ran et al., 2013). Single cell clones were grown by limiting dilution and then screened for ALC1 expression by western blot. For *in vivo* experiments, an YFP-labeled construct of ALC1 (YFP-ALC1 pDEST-YFP/FRT/TO) was used, previously generated by amplification from a human HeLa cDNA library and subsequent cloning by Gateway LR reaction (Ahel et al., 2009). ALC1 linker-mutations R611Q and R611A/S612A were introduced into ALC1 R860W YFP-ALC1 pDEST-YFP/FRT/TO (from Lehmann et al., 2017) by quick-change mutagenesis. Inducible YFP-ALC1 cell lines were generated using the Flp-In T-REx system (Invitrogen) as described in the manufacturer's protocol. Each YFP/FRT/TO construct was co-transfection with the pOG44 vector (Flp recombinase) into ALC1 −/− U2OS host cell lines. Recombination events were selected with 250 μg/ml hygromycin B (ThermoScientific).

DNA constructs

The DNA constructs used in the nucleosome assembly for single-molecule and ensemble remodeling studies comprised the Widom 601 nucleosome positioning sequence (Lowary and Widom, 1998) as well as either 3 bp or 12 bp of linker DNA on one side and 78 bp on the other side (for the single-molecule remodeling construct) or 63 bp of linker DNA on one side and no linker DNA on the other side (for the ensemble remodeling construct). All constructs were 5′-labeled with Cy5 on the short linker end. The constructs for single-molecule experiments additionally featured a biotin on the long linker end. DNA constructs were generated by PCR using HPLC-purified labeled oligonucleotide primers (IDT), followed by PAGE purification of the PCR product using a BioRad MiniPrep Cell or Prep Cell apparatus. The DNA constructs for the assembly of nucleosomes used in fluorescence anisotropy experiments and ATPase assays comprised the Widom 601 positioning sequence without additional linker DNA (for the fluorescence anisotropy construct) or 63 bp of linker DNA on one side and no linker DNA on the other side (for the ATPase assay construct). Both constructs were purified as described above.

Peptides

The following peptides were purchased from Peptide 2.0:

LANA peptide: MAPPGMRLRSGRSTGAPLTRGS
LANA peptide-TAMRA: MAPPGMRLRSGRSTGAPLTRGSK-/TAMRA/
ALC1^{RLS} peptide: EKASQEGRSLRNKGSVLIPGL
ALC1^{RLS} peptide-TAMRA: EKASQEGRSLRNKGSVLIPGLK-/TAMRA/
ALC1 linker peptide-Biotin: EKASQEGRSLRNKGSVLIPGLVEGSTKRKRVLSPEEK-/Biotin/

ALC1 expression and purification

All ALC1 constructs were expressed and purified as previously described (Lehmann et al., 2017). In brief, proteins were expressed in Rosetta 2 (DE3) cells (Novagen) and induced at 18°C overnight with 0.5 mM IPTG at OD_{600} = 2.0. For the purification, cell pellets were resuspended in Nickel column buffer A (20 mM HEPES pH 7.5, 500 mM NaCl, 20 mM imidazole, 10% glycerol, 5 mM β-mercaptoethanol), containing a protease inhibitor cocktail (Roche) and Benzonase Nuclease (Sigma). The cells were lysed using a sonicator, centrifuged, and the resulting supernatant was filtered through a 0.45 μm filter and purified over a 5 mL HisTrap FF column (GE Healthcare). Peak fractions were pooled and desalted using a HiPrep 26/10 desalting column (GE Healthcare) into S column buffer (20 mM MES pH 6.0, 300 mM NaCl, 10% glycerol, 1 mM DTT). The protein was loaded onto a 5 mL SP HP column (GE Healthcare), with a 5 mL Q HP column (GE Healthcare) attached in tandem to trap contaminating DNA, and eluted with a linear salt gradient. Peak fractions were injected onto a HiLoad 16/600 Superdex 200 pg column (GE Healthcare) equilibrated with 20 mM MES pH 6.5, 300 mM NaCl, 5% glycerol, and 0.5 mM TCEP. The final protein was concentrated to typically 10 mg/ml, flash frozen in liquid nitrogen, and stored at −80°C.

PARP1 expression and purification

Full-length human PARP1 was expressed and purified as previously described (Langelier et al., 2017). In brief, the PARP1 construct was introduced in Rosetta 2 (DE3) cells (Novagen) that were grown in the presence of 10 mM benzamide to OD_{600} = 0.8-1.0 at 37°C and after the addition of $ZnSO_4$ (final conc. 0.1 mM), expression was induced at 16°C overnight with 0.2 mM IPTG. For the purification, cell pellets were resuspended in resuspension buffer (25 mM HEPES pH 8, 500 mM NaCl, 0.5 mM TCEP, 10 mM benzamide and 0.1% NP-40), containing a protease inhibitor cocktail (Roche). The cells were lysed using a sonicator, centrifuged, and the resulting supernatant was filtered through a 0.22 μm filter and loaded on to a 5 mL HisTrap FF column (GE Healthcare) equilibrated with equilibration buffer (25 mM HEPES pH 8, 500 mM NaCl, 0.5 mM TCEP). After consecutive washes with Low Salt and High Salt Wash buffers (25 mM HEPES pH 8, 500 mM (low)/1 M (high) NaCl; 0.5 mM TCEP, 20 mM imidazole and protease inhibitor cocktail) protein was eluted using elution buffer supplemented with 400 mM imidazole. Peak fractions were pooled and diluted with No Salt Heparin buffer (50 mM Tris pH 7.0, 1 mM EDTA, 0.1 mM TCEP) to a final salt concentration of 375 mM NaCl prior to loading the protein onto a 5 mL HiTrap Heparin HP column (GE Healthcare) equilibrated with 375 mM NaCl containing Low Salt Heparin buffer. Protein was eluted using a linear salt gradient. Peak fraction were pooled, filtered through a 0.22 μm filter and loaded onto a HiPrep 16/60 Sephacryl S-200 HR (GE Healthcare) column equilibrated with gel filtration buffer (25 mM HEPES pH 8.0, 150 mM NaCl, 1 mM EDTA, 0.1 mM TCEP). The final protein was concentrated to 30 mg/ml and after flash freezing in liquid nitrogen stored at −80°C.

Histone expression and purification

Recombinant histones (H2A, H2A K120C, H2A APM, H2A APM K120C, H3 C110A and H4) from *Xenopus laevis* were purified essentially as described previously (Klinker et al., 2014). In brief, proteins were expressed in BL21 (DE3) pLysS (Novagen) cells and induced at OD_{600} = 0.6-0.8 for 2 h at 37°C with 1 mM IPTG. The cell pellets were resuspended in 40 mM NaOAc pH 5.2, 1 mM EDTA, 10 mM lysine, 200 mM NaCl, 5 mM β-mercaptoethanol, 6 M urea, and a protease inhibitor cocktail (Roche). Once homogenized, the cells were lysed using a sonicator, centrifuged, and the resulting supernatant was filtered through a 0.45 μm filter and purified over a 5 mL SP HP column (GE Healthcare), with a 5 mL Q HP column (GE Healthcare) attached in tandem to trap contaminating DNA and eluted with a salt gradient. Peak fractions were dialyzed overnight against cold water. The dialysate was mixed with Tris pH 8.0 to a final concentration of 15 mM and then passed over a 5 mL Q HP column (GE Healthcare). The final protein was concentrated to 5 mg/ml, flash frozen in liquid nitrogen, and stored at −80°C.

Histone labeling

Histones H2A K120C and H2A APM K120C were site-specifically labeled at cysteine residue 120 with Cy3-maleimide (GE Healthcare) as previously described (Yang et al., 2006; Blosser et al., 2009). In brief, one mg of histone protein was diluted in labeling buffer (20 mM Tris pH 7.0, 7 M guanidine-HCl, 5 mM EDTA, 1.25 mM TCEP) and incubated for 2 h at room temperature in the dark. Cy3-maleimide was dissolved in DMSO and added to the protein at a final concentration of 0.75 mM. After 3 h in the dark at room temperature, the reaction was quenched with a final concentration of 80 mM β-mercaptoethanol. The labeled protein was dialyzed nine times against dialysis buffer (20 mM Tris pH 7.0, 7 M guanidine-HCl, 1 mM DTT) and then used directly in an octamer assembly.

Histone octamer assembly

The four histones were combined at a molar ratio 1.2:1.2:1:1 (H2A:H2B:H3:H4), unfolded in 400 mM Tris pH 7.5, 7 M guanidine-HCl, 200 mM DTT and the histone octamer was assembled by dialyzing three times against refolding buffer (10 mM Tris-HCl pH 7.5, 2 M NaCl, 1 mM EDTA, and 5 mM β-mercaptoethanol). The assembly reaction was filtered through a 0.22 μm filter and loaded onto a HiLoad 16/600 Superdex 200 pg column (GE Healthcare) equilibrated with refolding buffer. Peak fractions were pooled, concentrated to 3 mg/ml, flash frozen in liquid nitrogen, and stored at −80°C.

Nucleosome assembly

Mononucleosomes were reconstituted from the DNA constructs and histone octamer (detailed above) by salt dialysis (Dyer et al., 2004). Nucleosomes for FRET-based assays were additionally subjected to PAGE purification using a BioRad MiniPrep Cell or Prep Cell apparatus.

ATPase activity measurements

ATPase activity was measured using a coupled assay that measures ADP production as described previously, (Willhoft et al., 2016). Final concentrations of 0.45 mM NADH, 1.0 mM phosphoenolpyruvate, 54 U/ml pyruvate kinase (Sigma), and 78 U/ml lactic dehydrogenase (Sigma) were used. For measurements of nucleosome-stimulated ATPase activity, reactions contained 20 μM ALC1, 2 μM WT/WT nucleosomes, and 1 mM ATP in a volume of 30 μl. Fluorescence was monitored (excitation wavelength: 340 nm; emission wavelength: 430 nm) at 37°C using a CLARIOstar microplate reader (BMG Labtech) and black low-volume 384-well microplates (Corning). For measurements of DNA-stimulated ATPase activity, the absorbance was monitored at 340 nm for reactions containing a final of 1 μM ALC1 and either 20 μM or 50 μM 145 bp nucleosomal double-stranded (ds) DNA in 100 μl. Reaction rates were determined using mean linear slopes. For measurements of nucleosome-stimulated ATPase activity, we estimated V_{max} for both ALC1^{fl} R860W and ALC1^{fl} R611A/S612A/R860W using the Michaelis-Menten equation and the K_m values that we obtained from nucleosome sliding kinetics in Figure 3B. Based on V_{max}, the difference in nucleosome-stimulated ATPase activity is ~2-fold lower for ALC1^{fl} R611A/S612A/R860W compared to ALC1^{fl} R860W (rather than ~2.5-fold).

Fluorescence anisotropy binding assay

For binding studies, we used nucleosomes without linker DNA (WT/WT or APM/APM) as well as the following synthetic peptides (as detailed above): LANA peptide-TAMRA, ALC1^{RLS} peptide-TAMRA, and ALC1^{RLS} peptide. Fluorescence anisotropy measurements were carried out at 30°C using a CLARIOstar microplate reader (BMG Labtech). Binding curves were recorded in black low-volume 384-well assay plates (Corning). Individual wells contained 10 nM peptide and the indicated concentration of nucleosomes in 15 mM HEPES pH 7.5, 1 mM EDTA, 5% glycerol, and 1 mM DTT. Dissociation constants were determined by fitting the fluorescence anisotropy data to the solution of a quadratic equation derived from the binding isotherm, which took into account depletion of peptide: $r = \alpha\left((A + K_D + B) - \sqrt{(A + K_D + B)^2 - 4AB}\right)$, where r is the fluorescence anisotropy, A represents the total concentration of peptide, α is a scaling factor, K_D is the dissociation constant, and B is the total concentration of titrated ligand. For competition binding experiments, WT/WT nucleosomes (100 nM) were incubated with LANA-TAMRA peptide (10 nM) prior to the addition of varying concentrations of unlabeled ALC1^{RLS} peptide.

Ensemble FRET assay for nucleosome remodeling

Nucleosome ensemble remodeling kinetics were measured by monitoring the Cy5 (under 620 nm and 520 nm excitation) and Cy3 (under 520 nm excitation) fluorescence emission signals of a solution of FRET-labeled nucleosomes using a Spark (Tecan) or CLARIOstar (BMG Labtech) multimode microplate reader. Ensemble nucleosome remodeling assays were performed with 10 nM nucleosomes, varying concentrations of ALC1 as indicated, 1 mM $MgCl_2$ and 1.5 mM ATP in remodeling buffer (20 mM HEPES pH 7.5, 50 mM KCl, 5 mM $MgCl_2$, 5% sucrose, 0.1 mg/ml BSA, 1 mM DTT). Where indicated, PARP1 was PARylated prior to nucleosome remodeling by incubating (in remodeling buffer at 37°C for 5 min) 80 nM or 5 μM PARP1 with 120 nM or 7.5 μM nucleosomal DNA as well as 50 μM or 100 μM NAD^+, for reactions with 80 nM or 5 μM ALC1, respectively. For remodeling assays in the presence of LANA peptide, 10 nM nucleosomes were incubated with 80 μM LANA peptide at room temperature for 5 min prior to the addition of $MgCl_2$ and ALC1. Remodeling rates were obtained as initial slopes of the remodeling curves. Reaction kinetics were analyzed assuming a standard Michaelis-Menten mechanism.

Single-molecule FRET remodeling

Using the DNA constructs for single-molecule FRET described above, we first generated oriented hexasomes with H2A-Cy3 on the short linker side using a salt dialysis method as described previously (Dyer et al., 2004; Levendosky et al., 2016; Sabantsev et al., 2019). Nucleosomes were then reconstituted by adding a 3-fold excess of WT or APM H2A/H2B (final concentration of dimer 1.2 μM) to purified hexasomes and incubating the mixture at 37°C for 10 min (Levendosky et al., 2016). This allowed for the controlled incorporation of the Cy3 label and AP mutations in a specific orientation (Figure 1D). Labeled nucleosomes were immobilized on a PEG (poly[ethylene glycol])-coated quartz slide using biotin-streptavidin interactions (Deindl et al., 2013). Cy3 and Cy5 fluorophores were excited with 532 nm Nd:YAG and 638 nm diode lasers, respectively, and fluorescence emissions from Cy3 and Cy5 were detected using a custom-built prism-based TIRF microscope. To check the presence of an intact donor fluorophore, the sample was alternately excited with 532 nm and 638 nm lasers during the experiment. Data acquisition was controlled using MicroManager (Edelstein et al., 2010). Data were analyzed using custom scripts for the Fiji distribution of ImageJ (Schindelin et al., 2012; Schneider et al., 2012), IDL, and MATLAB (Sabantsev et al., 2019). The initial remodeling direction was determined by visual inspection of the FRET traces. Remodeling experiments were carried out in the imaging buffer containing 40 mM Tris pH 7.5, 12 mM HEPES pH 7.9, 60 mM KCl, 0.32 mM EDTA, 3 mM $MgCl_2$, 100 μg/mL acetylated BSA (Promega), 10% (v/v) glycerol, 10% (w/v) glucose, 2 mM Trolox to reduce photoblinking of the dyes (Rasnik et al., 2006), as well as an enzymatic oxygen scavenging system (composed of 800 μg/ml glucose oxidase and 50 μg/ml catalase). Using a syringe pump (Harvard Apparatus), remodeling was initiated by infusing the sample chamber with imaging buffer supplemented with ALC1^{fl} R860W (2.5 μM), ATP (1 mM) and $MgCl_2$ (1 mM). The nucleosomes used for the quantification of remodeling directionality (Figure 1D) exhibited an intermediate starting FRET value of ~0.4. Upon remodeling, a subset of time traces displayed an intial FRET increase, followed by a subsequent FRET decrease after reaching the maximum

FRET value. Such a FRET signature could be caused by unidirectional remodeling that proceeds beyond the DNA end rather than by a change in remodeling direction. As soon as the end of the FRET dynamic range is reached (a maximum FRET value in this case), we cannot reliably distinguish between these two cases. For this reason, we limited our analyses to the initial portions of the time traces (before the edges of the FRET dynamic range are reached) that can be unambiguously interpreted.

Most traces exhibit repeated directional switching, which might be caused by two ALC1 molecules bound at opposing sides of the same nucleosome taking turns in remodeling it. Alternatively, such non-monotonous FRET changes could also be due to the consecutive binding of ALC1 molecules in different orientations, or stem from the same ALC1 molecule switching between the two opposing binding sites within a single binding event, as has been observed for Chd1 (Qiu et al., 2017). We note that our data do not allow us to distinguish between these scenarios.

The unusually long pauses observed for ALC1^{fl} R611A/S612A/R860W predominantly represent pauses in translocation by the same remodeler as opposed to separate remodeler binding events, based on the following considerations. First, the average pause duration was 2.8-fold shorter than the average binding time for the remodeler at a concentration of 20 μM. Second, lowering the remodeler concentration by a factor of 4 produced only a minor (30%) increase in the observed pause duration.

Cross-linking coupled to mass spectrometry

For cross-linking coupled to mass spectrometry, instead of ALC1^{fl} R860W we used ALC1^{fl} R857Q because that mutant could be produced in the required larger quantities. Importantly, biochemical and cell-based approaches have previously shown that both the R857Q and R860W mutations affect the same electrostatic interface and can be used interchangeably to render ALC1 constitutively active independent of PARP1 activation (Lehmann et al., 2017). ALC1^{fl} R857Q-nucleosome complexes were cross-linked by incubation with a 100-fold molar excess of N-hydroxysuccinimide (NHS) ester disuccinimidyl dibutyric urea (DSBU, formerly BuUrBu) for 45 min at room temperature. The reactions were quenched by adding NH_4HCO_3 to a final concentration of 50 mM and incubating for an additional 15 min. The cross-linked samples were freeze-dried and resuspended in 50 mM NH_4HCO_3, reduced with 10 mM DTT, and alkylated with 50 mM iodoacetamide. Following alkylation, proteins were digested with trypsin (Promega, UK) at an enzyme-to-substrate ratio of 1:20 overnight at 37°C or sequentially with trypsin and Glu-C (Promega, UK) at an enzyme-to-substrate ratio of 1:20 and 1:50 at 37°C and 25°C, respectively. The samples were acidified with formic acid to a final concentration of 2% (v/v), split into two equal amounts for peptide fractionation by peptide size exclusion chromatography (SEC) and reverse phase C18 high pH chromatography (C18-Hi-pH). For SEC, a Superdex Peptide 3.2/300 column (GE Healthcare) with 30% (v/v) acetonitrile/0.1% (v/v) TFA as the mobile phase and a flow rate of 50 μl/min were used, and fractions were collected every 2 min over an elution volume of 1.0 ml to 1.7 ml. C18-Hi-pH fractionation was carried out on an Acquity UPLC CSH C18 1.7 μm, 1.0 × 100 mm column (Waters) over a gradient of acetonitrile 2%–40% (v/v) and 100 mM NH_4HCO_3. Fractions were lyophilized and resuspended in 2% (v/v) acetonitrile and 2% (v/v) formic acid and analyzed by nano-scale capillary LC–MS/MS using an Ultimate U3000 HPLC (ThermoScientific Dionex, USA) to deliver a flow of approximately 300 nl/min. A C18 Acclaim PepMap100 5 μm, 100 μm × 20 mm nanoViper (ThermoScientific Dionex, USA) trapped the peptides before separation on a C18 Acclaim PepMap100 3 μm, 75 μm × 250 mm nanoViper (ThermoScientific Dionex, USA). Peptides were eluted with an acetonitrile gradient. The analytical column outlet was directly interfaced via a nano-flow electrospray ionisation source, with a hybrid quadrupole orbitrap mass spectrometer (Q-Exactive HF-X, ThermoScientific, USA). MS data were acquired in data-dependent mode. High-resolution full scans (R = 120,000, m/z 350-2000) were recorded in the Orbitrap followed by higher energy collision dissociation (HCD) (stepped collision energy 30 ± 3) of the 10 most intense MS peaks. MS/MS scans (R = 45,000) were acquired with a dynamic exclusion window of 20 s.

For data analysis, Xcalibur raw files were converted into the MGF format through MSConvert and used directly as input files for MeroX2. Searches were performed against an *ad hoc* protein database containing the sequences of the complexes and a set of randomized decoy sequences generated by the software. The following parameters were set for the searches: maximum number of missed cleavages 3; targeted residues K, S, Y and T; minimum peptide length 5 amino acids; variable modifications: carbamidomethyl-Cys (mass shift 57.02146 Da), Met-oxidation (mass shift 15.99491 Da); DSBU modification fragments: 85.05276 Da and 111.03203 (precision: 5 ppm MS1 and 10 ppm MS2); False Discovery Rate cut-off: 5%. Finally, fragmentation spectra were manually inspected and validated.

Cryo-EM sample preparation

A biotinylated ALC1 linker peptide was added to core nucleosomes (without any linker DNA on either side of the histone octamer) at 10-fold molar excess in the reaction buffer (15 mM HEPES pH 7.5, 1 mM EDTA, 1 mM DTT). After 10 min of incubation on ice, the complex was cross-linked with 0.05% glutaraldehyde for 5 min on ice. The cross-linking reaction was quenched by adding 1 M Tris-HCl pH 7.5 and the sample was dialyzed against purification buffer (15 mM HEPES 7.5, 150 mM NaCl, 1 mM EDTA, 5% glycerol, 0.01% NP-40, 1 mM DTT) for one hour at 4°C. After dialysis, the sample was applied onto a column custom-packed with Pierce Monomeric Avidin Agarose resin (Thermo Scientific) and incubated for 30 min to allow binding to the column. Washing and elution steps were performed with a controlled flow rate of 0.1 ml/min. The cross-linked complex between the nucleosome and biotinylated ALC1 linker peptide was eluted with the same purification buffer supplemented with 2 mM biotin. Fractions containing the complex were identified by native PAGE, pooled, concentrated, and buffer-exchanged using a 30 kDa cut-off spin concentrator in cryo-EM buffer (15 mM HEPES pH 7.5, 50 mM NaCl, 1 mM DTT). Quantifoil R 2/2 Cu 300 grids (Electron microscopy sciences) were glow-discharged at 20 mA for 60 s (PELCO easiGlow). 3 μl of the sample at 6.4 μM were applied onto grids and immediately blotted for

2.5 s. Sample application and blotting was repeated once more before rapid plunge-freezing into liquid ethane, using a Vitrobot Mark IV (Thermo Scientific) operated at 100% relative humidity and 4°C.

Cryo-EM data collection and processing

Cryo-EM data were collected at the Karolinska Institutet 3D-EM facility (Stockholm, Sweden) using a Titan Krios G3 microscope operated at 300 kV and equipped with a Gatan K3 Bioquantum detector and a GIF Quantum LS energy filter (slit width 20 eV). Movies were recorded in counting mode, gain-corrected, at a calibrated pixel size of 0.654 Å/pixel, with a total exposure of 50.4 electrons/$Å^2$ fractionated over 60 movie frames (resulting in an exposure per frame of 0.84 electrons/$Å^2$/frame). Motion correction, CTF estimation and particle picking were performed during data collection using Warp (Tegunov and Cramer, 2019). A subset of the particles extracted by Warp were imported into cryoSPARC (Punjani et al., 2017) for evaluation by 2D and 3D classification. The entire dataset was then reprocessed in RELION-3.1.0 (Scheres, 2012; Zivanov et al., 2018, 2020). Motion correction was performed using MotionCor2 version 1.3.1 (Zheng et al., 2017) and CTF estimation was performed using Gctf version 1.18b2 (Zhang, 2016), both from within RELION. Particle coordinates from Warp were imported into RELION. Unsuitable particles were excluded after one round of reference-free 2D classification. The initial 3D model was generated from the data using the InitialModel (stochastic gradient descent) procedure in RELION. Unsuitable particles were further excluded in six rounds of 3D classification, without any symmetry constraints applied (C1). Round 3 of 3D classification identified two sets of particles corresponding to a nucleosome with both APs occupied by a peptide and a hexasome with its single AP occupied by a peptide. These two classes were independently subjected to 3D refinement, CTF refinement (in three successive steps: beam tilt and higher-order aberrations, anisotropic magnification, and per-particle defocus plus per-micrograph astigmatism), 3D refinement, Bayesian polishing and a final 3D refinement (Figure S2; Table S2). Consistent with complex preparation by first saturating the nucleosome with ALC1 linker peptide, followed by crosslinking and purification of nucleosomes-peptide complexes, all nucleosome particles had two peptides bound, occupying the APs on both sides of the nucleosome. The dataset contains neither unbound nucleosomes, nor nucleosomes with only one AP occupied. Our dataset also contains hexasomes (nucleosomes lacking one H2A-H2B dimer), since partial nucleosome disassembly upon vitrification is a common phenomenon (Bilokapic et al., 2018). Interestingly, the hexasome class also displayed density from the bound ALC1 linker peptide on the remaining H2A/H2B dimer, supporting our *in vitro* characterization of cross-linking efficiency: because the affinity of the peptide for the AP is much lower than that of the H2A/H2B dimer for the hexasome, we would not expect to see density for the peptide under conditions that lead to dissociation of one H2A/H2B dimer, yet we observe such density here, consistent with efficient cross-linking. The local resolution was estimated with RELION's local Fourier Shell Correlation procedure.

Model building and refinement

We built a model of a nucleosome with both APs bound by the ALC1 linker peptide from PDB entries 3LZ0 (nucleosome with *Xenopus laevis* histones and Widom 601 DNA) (Vasudevan et al., 2010) and 1ZLA (LANA peptide) (Barbera et al., 2006) and performed rigid-body fitting of this model into our cryo-EM map using UCSF ChimeraX version 0.93 (Goddard et al., 2018). The resolution of our maps allowed us to distinguish between the two asymmetric sides of the 601 sequence, so we oriented the nucleosome model properly into the map in the orientation that gave the best model-to-map real-space correlation coefficient. We edited the LANA peptide sequence to match the ALC1 linker peptide and deleted residues not supported by density in our cryo-EM map, using Coot version 0.9-pre-855 (Casañal et al., 2020). We finally performed flexible fitting of the model into the cryo-EM map using ISOLDE version 1.0b5 (Croll, 2018). The resulting refined model was then rigid-body fit into the hexasome map, the H2A, H2B and ALC1 linker peptide chains were deleted from the model on the side of the hexasome map devoid of density for these chains, DNA nucleotides not supported by density were also deleted from the model (corresponding to the more easily unwrapped side of the 601 sequence), and the resulting hexasome model was subjected to flexible fitting in ISOLDE. For more details on the model building, see also Supplemental Methods. Model-to-map real space correlation coefficients and model geometry statistics were calculated with phenix.validation_cryoem, from the Phenix suite version 1.18.2-3874 (Liebschner et al., 2019). Model validation statistics are presented in Table S2.

Whole-cell extracts and immunoblotting

For whole cell lysates, PBS washed cells were lysed in RIPA Buffer (10 mM Tris-Cl pH 8.0, 1 mM EDTA, 0.5 mM EGTA, 1% Triton X-100, 0.1% sodium deoxycholate, 0.1% SDS, 140 mM NaCl, 1x phosphatase mix (Phos-Stop, Roche) and complete EDTA-free protease inhibitor mix (Roche)) on ice for 20 min. Lysates were sonicated with a probe at medium intensity for 5 s in a Soniprep 150 instrument and clarified by centrifugation at 13000 xg for 10 min at 4°C. Proteins were denatured in 2X NuPAGE LDS sample buffer (Invitrogen) and 1% 2-metcaptoethanol (Sigma-Aldrich) for 5 min at 95°C. Proteins were separated by SDS-PAGE using NuPAGE mini gels (Invitrogen) and transferred onto 0.4 PVDF (Millipore). Membranes were blocked with 5% skim milk/PBST (PBS/0.1% Tween-20) for 1 h at room temperature and probed with ALC1 1:1000 (Cell Signaling Technology), hnRNPA1 1:3000 (Sant Cruz Biotechnology) primary antibodies overnight at 4°C. Membranes were then washed 3 times for 10 min with PBST, incubated with appropriate secondary antibodies conjugated to a horseradish peroxidase (HRP) for 1 h at room temperature and washed again 3 times for 10 min with PBST. Immunoblots were developed using Clarity or Clarity Max Western ECL Substrate (Bio-Rad).

Laser damage

Cells were seeded on 8 well Lab-Tek chamber slides (Thermo Fisher Scientific). Cells were pre-sensitized with 10 μM BrdU and treated with 1 μg/ml doxycycline 24 hours prior to imaging. Cells were transferred to an Nikon Ti-E with: Andor FRAPPA unit, Yokogawa CSU-X spinning disk scanhead, Andor iXon 897 EM-CCD camera with a heat and atmosphere controlled incubator from OKO labs. Laser micro-irradiation was performed with a 405 nm laser focused through a 60x 1.45 NA TIRF lens. Laser power was set to 50% and regions of interest (ROIs) were bleached for 20 iterations. In order to monitor the association kinetics of YFP-tagged ALC1 at sites of laser micro-irradiation, the time evolution of YFP fluorescence in the damaged region of interest was recorded upon excitation with a 488 nm laser. Recruitment of YFP to bleached ROIs was analyzed in FIJI (Schindelin et al., 2012) and data were processed in R. In brief, nuclear masks were segmented by local thresholding. FRAPped nuclei were identified by overlap of the nuclear mask with a bleached ROI. Nuclei with a circularity score less of than 0.4 were excluded from the analysis or if they contained multiple ROIs. In order to quantify recruitment, the mean YFP fluorescence intensities at a given time point t (I_t) were normalized to the mean initial fluorescence intensities I_0 of the same region of interest. Normalized intensities were additionally multiplied with BGI_0/BGI_t to correct for photobleaching, where BGI represents the background fluorescence intensity. In order to compare association rates, the time course of fractional recruitment was determined as $(I_t - I_0)/(I_{max} - I_0)$. Association kinetics were fit with a single-exponential (one-phase association) to obtain half-time constants.

QUANTIFICATION AND STATISTICAL ANALYSIS

For fluorescence anisotropy binding experiments, the reported values represent the mean ± SEM from 3 independent experiments. For ATPase activity measurements, the reported values represent the mean ± standard deviation from 3 independent experiments. For ensemble nucleosome remodeling time courses, each experimental condition was recorded in 3 independent experiments, and representative remodeling time courses are shown. Relative remodeling rates derived from these curves represent the mean ± standard deviation from 3 independent experiments. For smFRET experiments, values are reported as mean ± SEM from several experiments as specified in the corresponding figure legends. For association kinetics from live-cell imaging, error bars represent SEM from N $\geq$ 131 traces from 3 independent experiments.

Cell Reports, Volume *33*

Supplemental Information

Mechanistic Insights into Regulation of the ALC1 Remodeler by the Nucleosome Acidic Patch

Laura C. Lehmann, Luka Bacic, Graeme Hewitt, Klaus Brackmann, Anton Sabantsev, Guillaume Gaullier, Sofia Pytharopoulou, Gianluca Degliesposti, Hanneke Okkenhaug, Song Tan, Alessandro Costa, J. Mark Skehel, Simon J. Boulton, and Sebastian Deindl

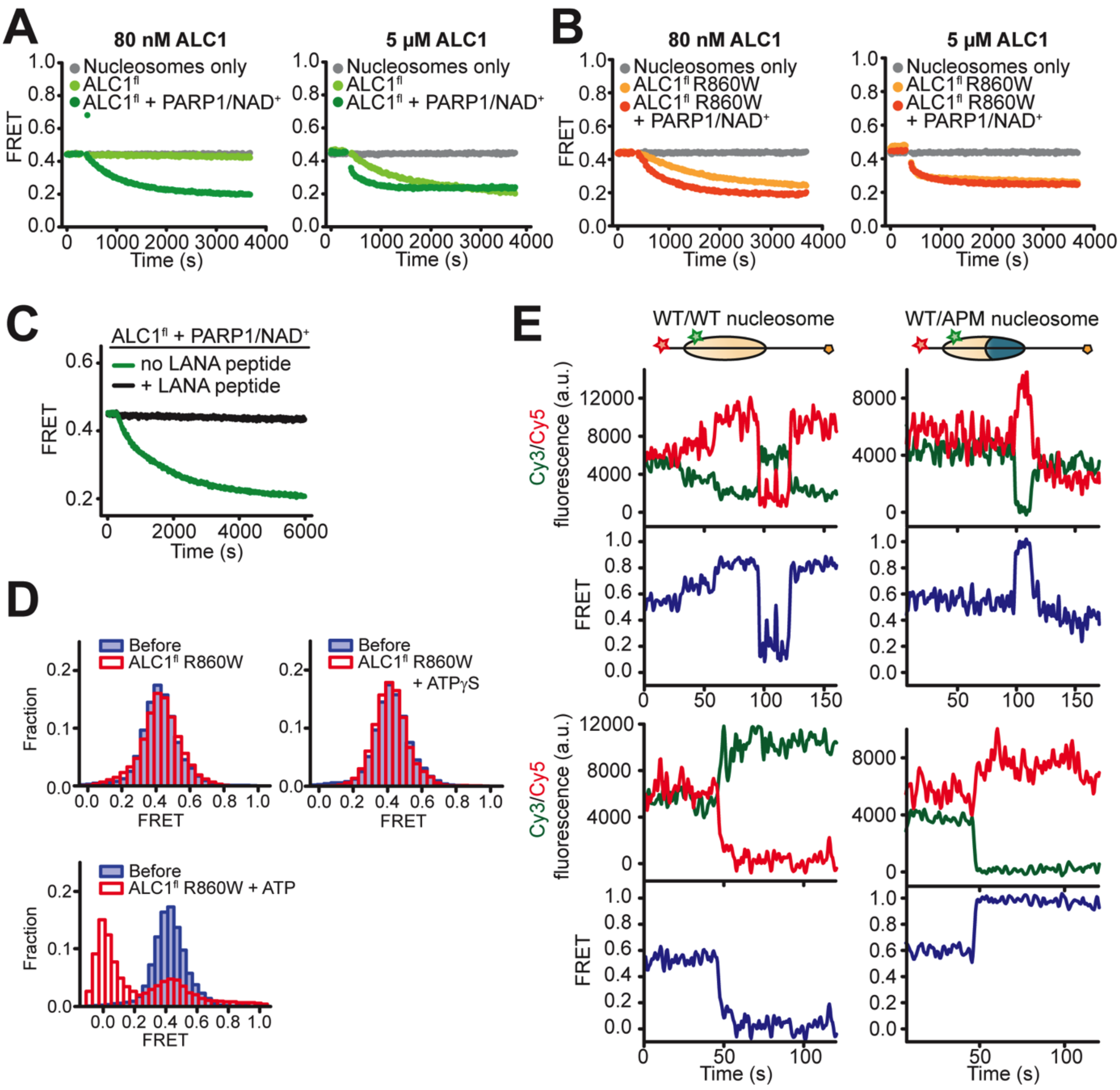

Figure S1. Related to Figure 1. The acidic patch is important for ALC1 remodeling.
(**A**) Ensemble remodeling time courses of initially end-positioned WT/WT nucleosomes (10 nM) alone (grey) or with 80 nM (left) or 5µM (right) ALC1^{fl} with (dark green) or without (light green) 80 nM PARP1 and 50 µM NAD^{+}. Representative curves from $N = 3$ independent experiments are shown. (**B**) Ensemble remodeling time courses of initially end-positioned WT/WT nucleosomes (10 nM) alone (grey) or with 80 nM (left) or 5µM (right) ALC1^{fl} R860W with (dark orange) or without (light orange) 80 nM PARP1 and 50 µM NAD^{+}. Representative curves from $N = 3$ independent experiments are shown. (**C**) Ensemble remodeling time courses of initially end-positioned WT/WT nucleosomes (10 nM) by 80 nM ALC1^{fl}, activated with 80 nM PARP1 and 50 µM NAD^{+}, in the presence (black) and absence (dark green) of a peptide derived from the N-terminal portion of the latency associated nuclear antigen (LANA) protein of the Kaposi's sarcoma-associated herpesvirus (KSHV). Representative curves from $N = 3$ independent experiments are shown. (**D**) Single-molecule FRET histograms for WT/WT nucleosomes before (blue) and after (red) the addition of ALC1^{fl} R860W alone (top left), with ATPγS (top right), or with ATP (bottom). (**E**) Representative donor (green) and acceptor (red) intensity (top), as well as corresponding smFRET time traces (blue, bottom) showing the ALC1^{fl} R860W-catalyzed remodeling of individual WT/WT (left) or WT/APM (right) nucleosomes.

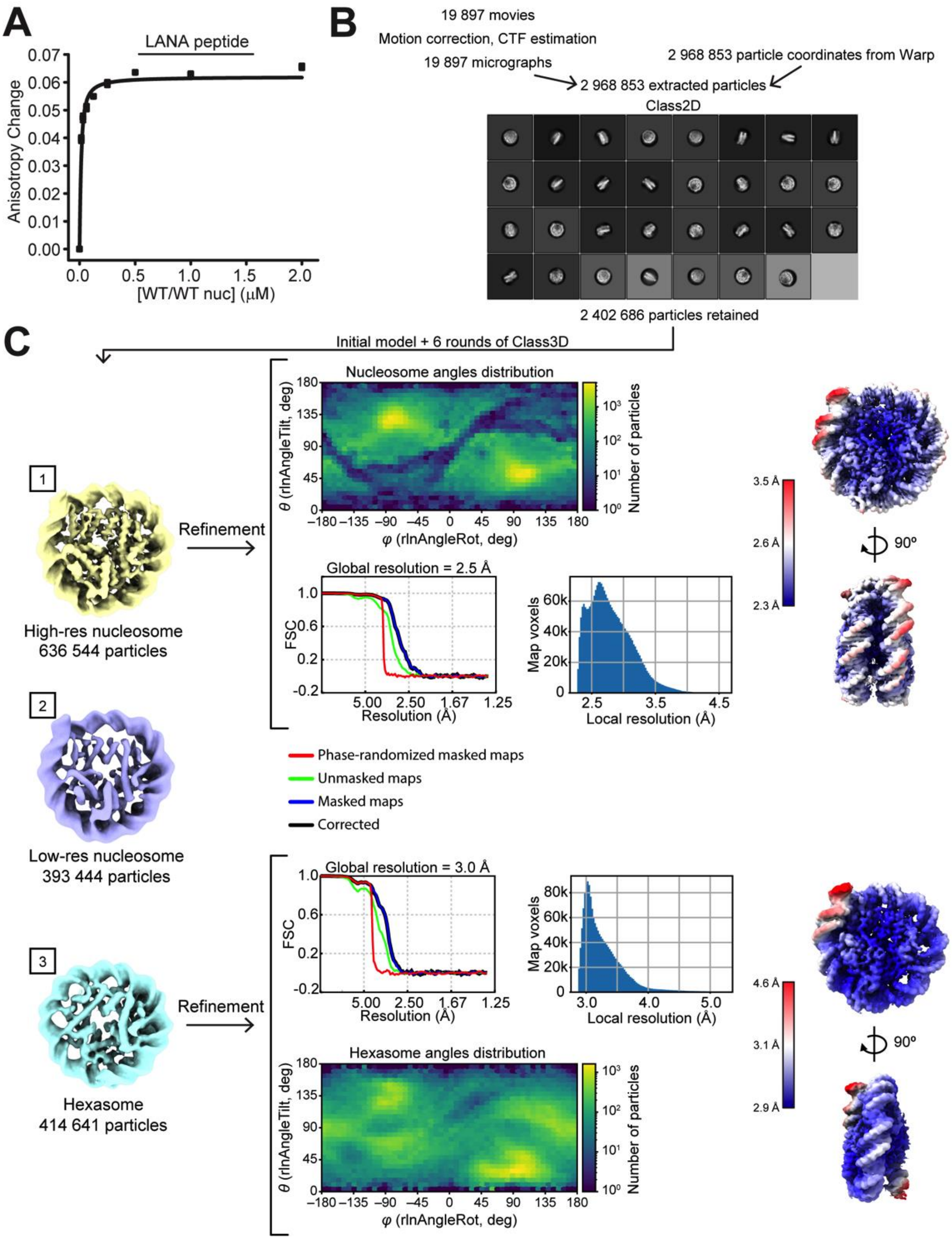

Figure S2. Related to Figure 2. LANA binding to nucleosomes and flowchart for cryo-EM data collection. (**A**) Fluorescence anisotropy binding measurements with TMR-labeled LANA peptide and varying amounts of WT/WT nucleosomes. The dissociation constant (K_d) is 9 nM. Error bars: S.E.M. (N = 3 independent experiments). (**B**) Suitable particles for cryo-EM structure determination were identified by one round of 2D classification followed by three rounds of 3D classification. (**C**) 3D classification identified three classes corresponding to (1) a high-resolution nucleosome, (2) a lower resolution nucleosome, and (3) a hexasome. The high-resolution nucleosome and hexasome classes were further subjected to three more rounds of 3D classification, yielding subsets of homogeneous particles suitable for refinement. Refinement protocol: 3D auto-refinement, three CTF refinements (beam tilt, anisotropic magnification, per-particle defocus and per-micrograph astigmatism), one 3D auto-refinement, Bayesian polishing, and a final 3D auto-refinement.

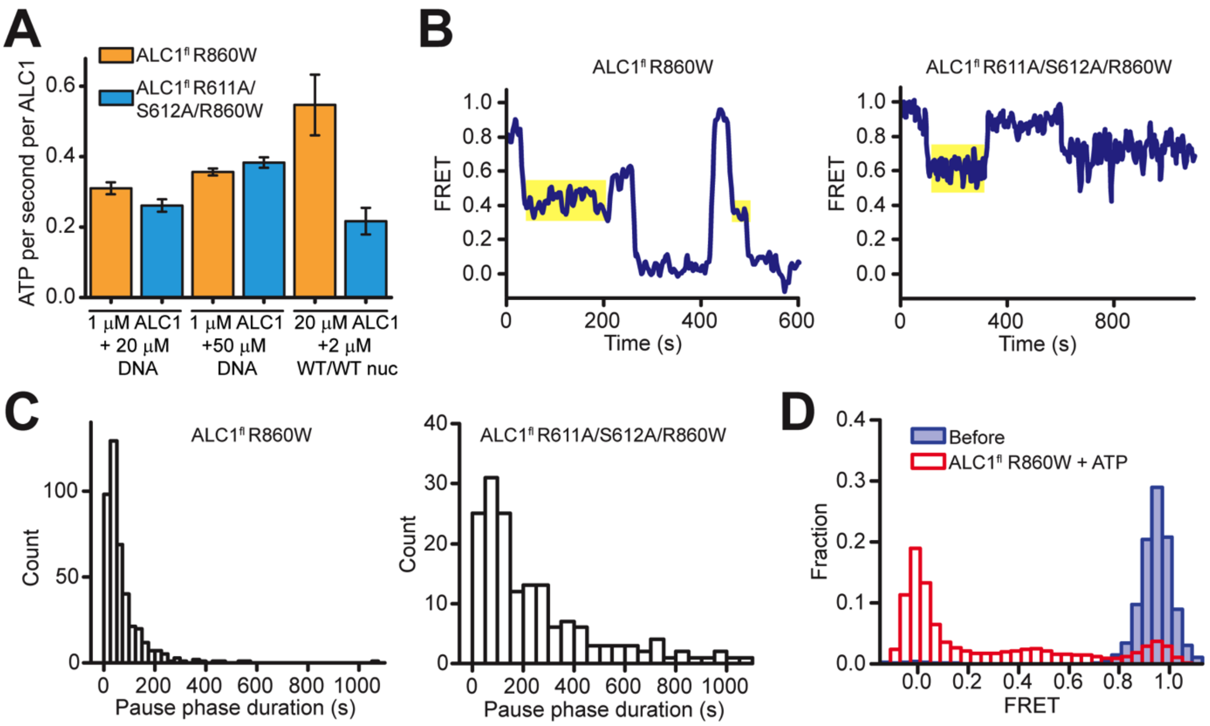

Figure S3. Related to Figure 3. Mutations in the regulatory linker segment of ALC1 affect the pause phase duration during nucleosome remodeling.
(**A**) ATPase rates for 1 µM ALC1^{fl} R860W (orange) or ALC1^{fl} R611A/S612A/R860W (light blue) in the presence of 20 µM or 50 µM dsDNA and 1 mM ATP. For comparison, the ATPase rates for 20 µM ALC1^{fl} R860W or ALC1^{fl} R611A/S612A/R860W in the presence of 2 µM WT/WT nucleosomes and 1 mM ATP are shown (same as shown in Figure 3A). Error bars represent standard deviation (N = 3 independent experiments). (**B**) Representative single-molecule FRET time traces showing the remodeling of individual WT/WT nucleosomes with 3 and 78 bp of flanking linker DNA by ALC1^{fl} R860W (left) and by ALC1^{fl} R611A/S612A/R860W (right). Pause phases are highlighted in yellow. (**C**) Histograms of pause phase durations derived from single-molecule FRET time traces for ALC1^{fl} R860W (left) and ALC1^{fl} R611A/S612A/R860W (right) (N = 421 or 171 plateaus from 166 or 220 time traces for ALC1^{fl} R860W or ALC1^{fl} R611A/S612A/R860W, respectively). (**D**) Single-molecule FRET histograms for WT/WT nucleosomes before (blue) and after (red) the addition of ALC1^{fl} R860W and ATP.

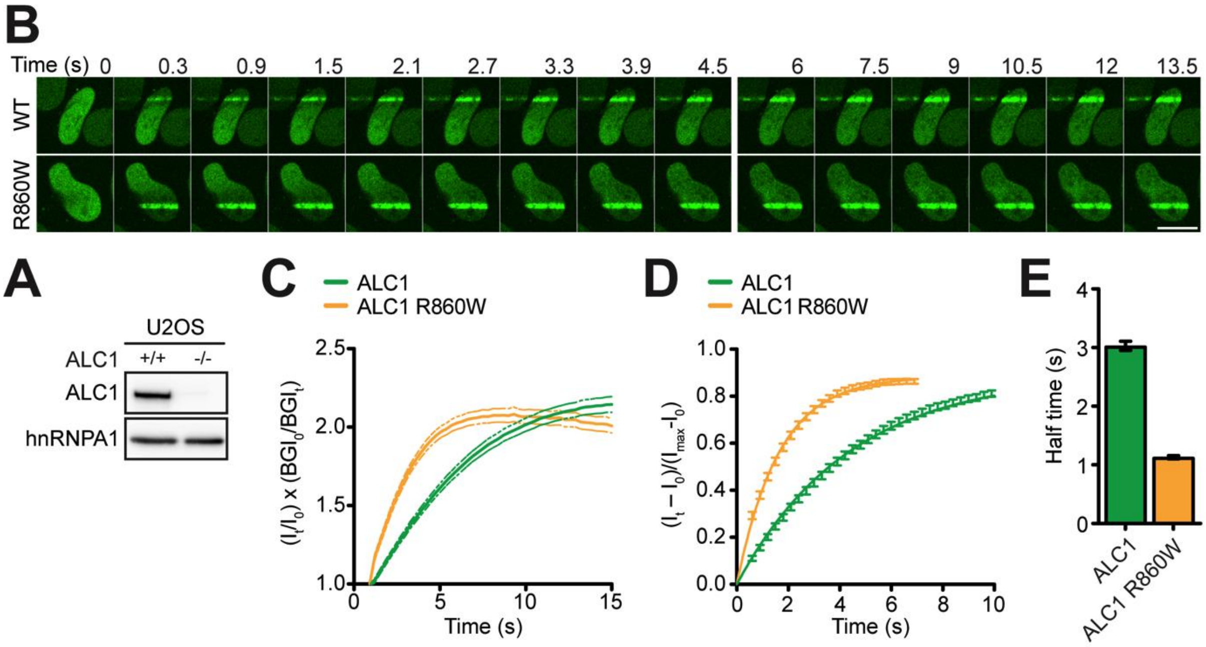

Figure S4. Related to Figure 4. Kinetics of YFP-ALC1 and YFP-ALC1 R860W association with DNA breaks.

(**A**) CRISPR-mediated inactivation of ALC1 in U2OS cells. Immunoblot of whole cell extracts from $ALC1^{+/+}$ and $ALC1^{-/-}$ cells, probed for ALC1. Heterogeneous Nuclear Ribonucleoprotein A1 (hnRNPA1) was probed for as a loading control. (**B)** Representative images of U2OS cells expressing WT or R860W YFP-ALC1, recorded at the specified time points upon laser damage. Scale bar: 10 µm. (**C)** Kinetics of WT (green) and R860W (orange) YFP-ALC1 association with DNA breaks. Fluorescence intensities were normalized and corrected for photobleaching (see Methods section). Error bars represent S.E.M. ($N \geq 131$ traces from 3 independent experiments). (**D**) Fraction of maximal recruitment of WT (green) and R860W (orange) YFP-ALC1 to DNA breaks. Error bars represent S.E.M. ($N \geq 131$ traces from 3 independent experiments). Solid line represents one-phase association. (**E**) Half-time quantified from (D). Data are mean ± 95% confidence intervals.

Protein 1	Position 1	Protein 2	Position 2
ALC1^{fl} R857Q	130	H3	5
ALC1^{fl} R857Q	130	H3	28
ALC1^{fl} R857Q	141	H3	5
ALC1^{fl} R857Q	141	H3	15
ALC1^{fl} R857Q	141	H3	19
ALC1^{fl} R857Q	141	H3	28
ALC1^{fl} R857Q	144	H3	5
ALC1^{fl} R857Q	144	H3	10
ALC1^{fl} R857Q	144	H3	19
ALC1^{fl} R857Q	144	H3	28
ALC1^{fl} R857Q	310	H3	80
ALC1^{fl} R857Q	320	H4	60
ALC1^{fl} R857Q	605	H2B	106
ALC1^{fl} R857Q	605	H2B	113
ALC1^{fl} R857Q	605	H2B	118
ALC1^{fl} R857Q	605	H2B	123
ALC1^{fl} R857Q	605	H4	9
ALC1^{fl} R857Q	607	H2B	123
ALC1^{fl} R857Q	612	H3	28
ALC1^{fl} R857Q	616	H2B	106
ALC1^{fl} R857Q	630	H2A	96
ALC1^{fl} R857Q	630	H2B	106
ALC1^{fl} R857Q	630	H2B	123
ALC1^{fl} R857Q	630	H3	80
ALC1^{fl} R857Q	630	H4	78
ALC1^{fl} R857Q	653	H3	28
ALC1^{fl} R857Q	661	H4	45
ALC1^{fl} R857Q	662	H3	15
ALC1^{fl} R857Q	757	H3	4
ALC1^{fl} R857Q	757	H3	5
ALC1^{fl} R857Q	757	H3	19
ALC1^{fl} R857Q	793	H2A	6
ALC1^{fl} R857Q	793	H3	5
ALC1^{fl} R857Q	793	H3	10
ALC1^{fl} R857Q	826	H3	19
ALC1^{fl} R857Q	848	H2A	96
ALC1^{fl} R857Q	848	H2B	106
ALC1^{fl} R857Q	848	H2B	118
ALC1^{fl} R857Q	848	H3	57

Table S1. Related to Figure 2. List of crosslinks between ALC1^{fl} and histone core proteins.
Cross-links to the ATPase domain of ALC1 (Protein 1) are shown in light green, to the linker region in magenta, and to the macro domain in dark blue. Crosslinks to the core histones (Protein 2) are shown in yellow for H2A, red for H2B, dark green for H3, and light blue for H4.

	Nucleosome class (EMD-11220, PDB 6ZHX)	Hexasome class (EMD-11221, PDB 6ZHY)
**Data collection and processing**		
Acceleration voltage (kV)	300	
Spherical aberration (mm)	2.7	
Amplitude contrast (fraction)	0.1	
Image pixel size (Å/pixel)	0.654	
Electron exposure per frame (e^-/Å^2)	0.84	
Number of movie frames	60	
Total electron exposure (e^-/Å^2)	50.4	
Nominal defocus range (µm)	-1 to -3	
Number of movies collected	19 897	
Number of picked particles	2 968 853	
Particles used for reconstruction	636 544	414 641
Map symmetry imposed	C1	
Map sharpening B-factor (Å^2)	-10	
Global resolution at 0.143 FSC (Å)	2.5	3.0
Local resolution range (Å)	2.3 - 3.5	2.9 - 4.6
**Model building and refinement**		
Initial models used (PDB codes)	3LZ0, 1ZLA	
Number of atoms (hydrogens)	21 581 (9 625)	16 141 (7 186)
Number of protein residues	759	557
Number of DNA residues	290	220
Bond length RMSD (Å)	0.014	0.014
Bond angles RMSD (º)	2.062	2.103
MolProbity score	0.62	0.55
Clash score	0.23	0.12
Ramachandran outliers/allowed/favored (%)	0.00 / 1.62 / 98.38	0.00 / 1.66 / 98.34
Rotamer outliers (%)	1.11	0.65
Cα outliers (%)	0.28	0.58
CαBLAM outliers (%)	0.56	0.76
Model to map CC (mask/peaks/volume)	0.78 / 0.68 / 0.78	0.80 / 0.74 / 0.80

Table S2. Related to Figure 2. Cryo-EM data collection, data processing, model building and model refinement, and validation statistics.

Mutations/ Constructs	Forward Primers	Reverse Primers
H2A E61A /E64A /D90A/E92A	Step 1 GTATCTGACCGCTGCGATTTTGGA ATTGGC Step 2 ACCGCTGCGATTTTGGCGTTGGCC GGGAAT Step 3 AGCTCGCTGTGCGCAACGCGGAG GCACTGAACAAACTGCTCGGAA	Step 1 GCCAATTCCAAAATCGCAGCGGTCA GATAC Step 2 ATTCCCGGCCAACGCCAAAATCGCA GCGGT Step 3 TTCCGAGCAGTTTGTTCAGTGCCTCC GCGTTGCGCACAGCGAGCT
ALC1^{fl} R611A/S612A /R860W	AAAGCGAGCCAGGAAGGTGCGGC GCTGCGTAATAAAGGCTCTG	CAGAGCCTTTATTACGCAGCGCCGC ACCTTCCTGGCTCGCTTT
ALC1^{fl} R611Q/R860W	CGAGCCAGGAAGGTCAGTCTCTG CGTAATAAAG	CTTTATTACGCAGAGACTGACCTTCC TGGCTCG
ALC1 R611A/S612A /R860W	GAAAGCTAGTCAAGAGGGCGCTG CACTCCGAAATAAAGGCAG	CTGCCTTTATTTCGGAGTGCAGCGCC CTCTTGACTAGCTTTC
ALC1 R611Q/R860W	GCCAGGAAGGTCAGTCTCTGCGT AATAAAGGC	GCCTTTATTACGCAGAGACTGACCT TCCTGGC
63-601-0-Cy5 nucl. DNA	CGCTGTTTTCGAATTTACCC	/5Cy5/ATCAGAATCCCGGTGCCG
63-601-0 nucl. DNA	CGCTGTTTTCGAATTTACCC	ATCAGAATCCCGGTGCCG
0-601-0 nucl. DNA	ATCGATGTATATATCTGACACGTG CC	ATCAGAATCCCGGTGCCG

Table S3. Related to STAR Methods. List of DNA oligonucleotides that were used in this study.

Methods S1. Related to STAR Methods. Details on the structural modeling.

The ALC1-linker peptide used to form a complex with the nucleosome for structure determination by cryo-EM has the following sequence:

```
EKASQ E  G  R  S  LRNKGSVLIPGLVEGSTKRKRVLSPEEK-Biotin
      -2 -1  0 +1 +2
```

The experimental density in our cryo-EM map can accommodate four residues (Figure M1, Figure M2), with a well-defined arginine side chain density ('Arg anchor') being the most salient feature of the map.

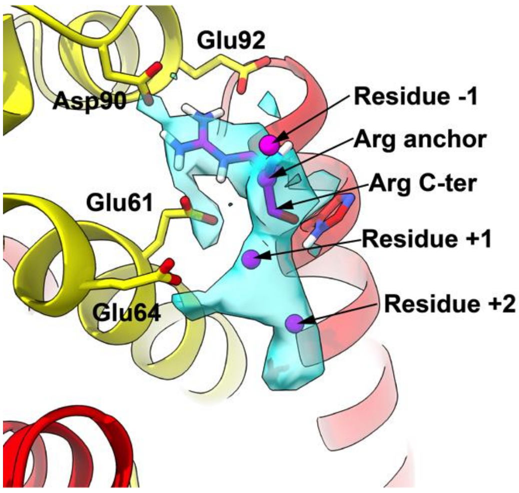

Figure M1. Arg anchor residue (sticks) and Cα atoms of one residue upstream and two residues downstream (spheres).

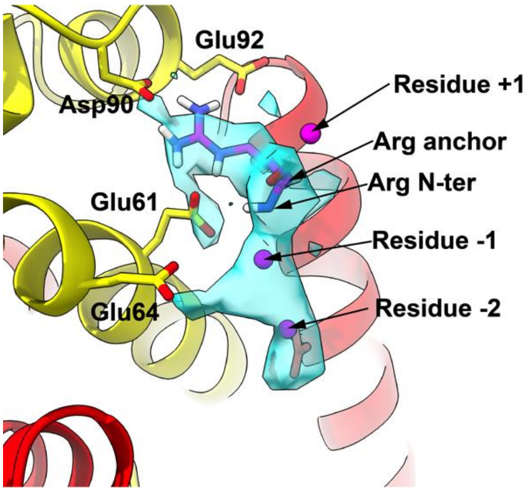

Figure M2. Arg anchor residue (sticks) and Cα atoms of two residues upstream and one residue downstream (spheres).

Since the ALC1-linker peptide contains three other arginine residues in addition to R611, we considered all of them when building our model (Figure M1):

Segment A: GRSL (highlighted in green in the sequence above)
Segment B: LRNK (underlined, overlapping with Segment A in green)
Segment C: KRKR (highlighted in red)
Segment D: KRVL (underlined, overlapping with Segment C in red)

In addition to these four segments with the arginine anchor at position 2, we also examined the following 4 segments with the arginine anchor at position 3, corresponding to the four segments above but modeled in the opposite N-terminal to C-terminal direction (Figure M2):

Segment E: EGRS
Segment F: SLRN
Segment G: TKRK
Segment H: RKRV

We built each of these eight four-residue segments and individually subjected them to real-space refinement against the cryo-EM map using the molecular dynamics flexible fitting (MDFF) engine implemented in ISOLDE (Croll, 2018). This approach not only aims to optimize the fit of the model to the map, but also imposes physically allowed conformations and electrostatic interactions in the model, with an automatically determined weighting factor between the map and the molecular dynamics force field. Therefore, the resulting

model is necessarily a compromise between these two optimization targets, and an optimal model may not exhibit a perfect fit to the map, since all cryo-EM maps intrinsically feature experimental measurement errors. Importantly, the resulting model features energetically and sterically allowed conformations.

The figures below show the result of such real-space refinement for each of the eight four-residue segments and follow the same color code as the one used in the manuscript: yellow for histone H2A, red for histone H2B, magenta for the ALC1-linker and translucent blue for the experimental cryo-EM density. In all figure legends, the sequence of each segment is indicated and the arginine residue occupying the “anchor” position is underlined.

Segment C (K<u>R</u>KR) does not fit the density, with a lysine residue too bulky to be accommodated without steric clashes in the space between the peptide backbone and residue Glu64 of H2A (Figure M3). Although this lysine was built pointing straight at H2A-Glu64, MDFF promptly moved it away. Modeling this segment with the opposite N-terminal to C-terminal polarity (Segment G) also places a lysine at this location, and therefore results in a similarly poor fit to the map (Figure M4).

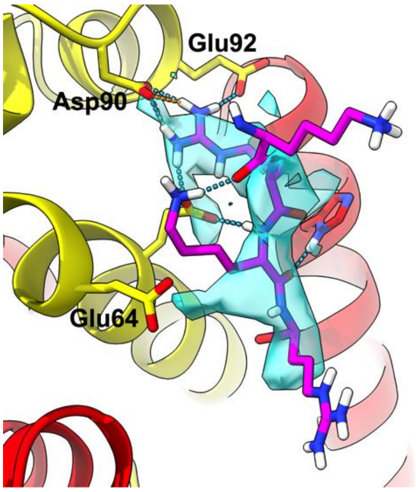

Figure M3. Segment C: K<u>R</u>KR

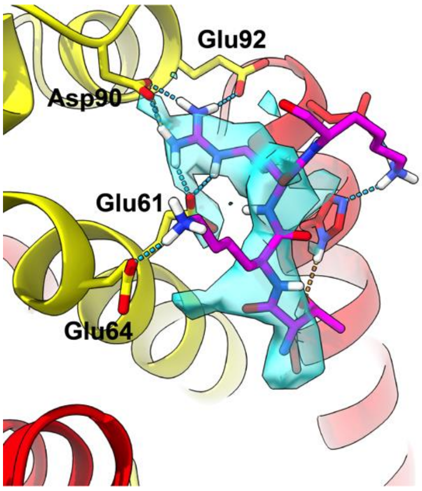

Figure M4. Segment G: TK<u>R</u>K

Segment D (K<u>R</u>VL) does not fit the density and does not make sense chemically with the hydrophobic valine residue facing the charged residue Glu64 of H2A, which is less favored than a hydrogen-bond donor (Figure M5). Modeling the opposite N-terminal to C-terminal polarity (Segment H) places a lysine at this location which, as we showed in Figure M3 and M4 above, does not fit the density either (Figure M6).

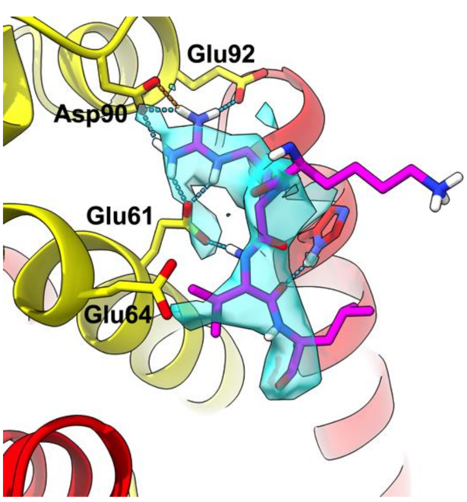

Figure M5. Segment D: K<u>R</u>VL

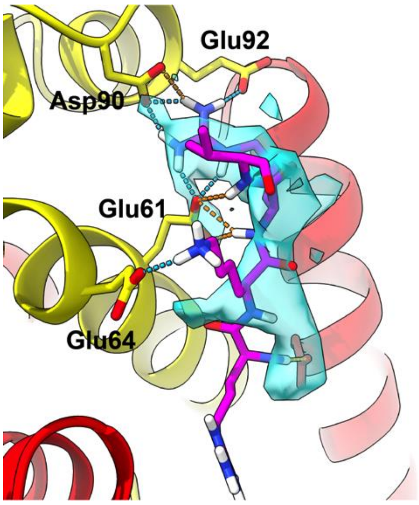

Figure M6. Segment H: RK<u>R</u>V

Segment B (LRNK) has an asparagine residue able to establish a hydrogen-bond with residue Glu64 of H2A (Figure M7), but this residue does not fit the density as well as the serine residue in Segment A (GRSL, Figure M9; see also Table M1). Modeling the opposite N-terminal to C-terminal polarity (Segment F) places a leucine in front of residue Glu64 of H2A, which is too bulky to be accommodated in this space and also does not make sense chemically since it cannot establish a hydrogen bond with H2A-Glu64 which, as a result, gets pushed away and no longer satisfactorily explains the density at its location (Figure M8).

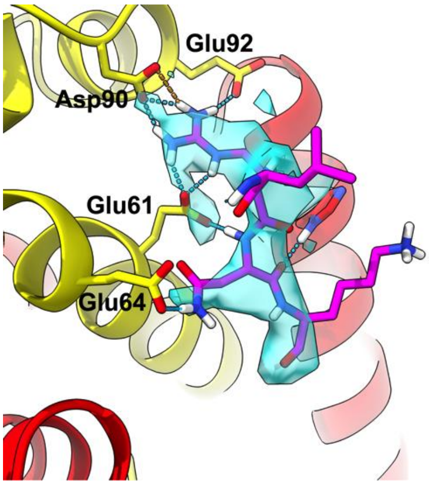

Figure M7. Segment B: LRNK

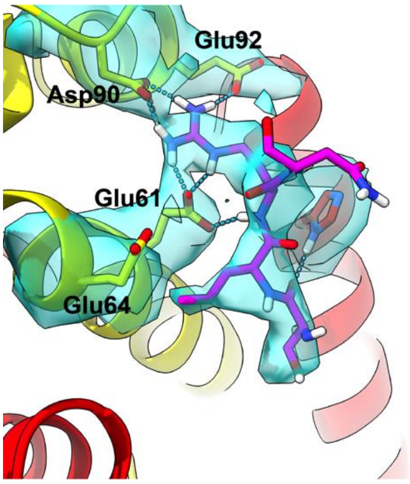

Figure M8. Segment F: SLRN
The map is displayed at the same contour level as in other figures, but also displayed around histone residues to show the poor fit of H2A-Glu64.

Segment A (GRSL) explains the observed density better than all the models shown above and establishes a more favorable hydrogen-bond network with histones than most of the other models (Figure M9). Modeling the opposite N-terminal to C-terminal polarity (Segment E) places a glycine in front of residue Glu64 of H2A, which does not explain the observed density, since glycine has no side chain (Figure M10), and leaves Glu64 of H2A without a hydrogen-bond donor.

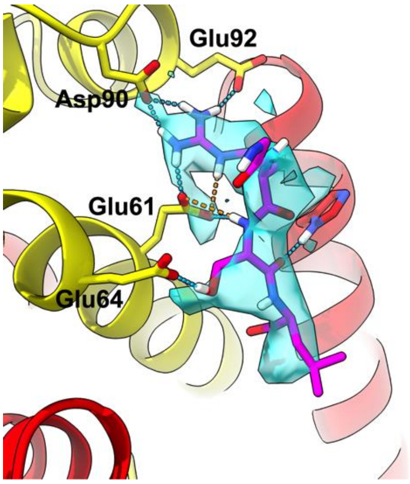

Figure M9. Segment A: GRSL

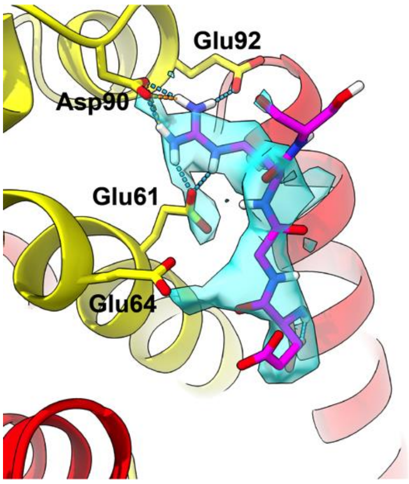

Figure M10. Segment E: EGRS

Clearly, all models with an N-terminal to C-terminal directionality opposite to Segment A display extremely poor fits to the map (Figures M4, M6, M8 and M10). Among the models with correct N-terminal to C-terminal directionality (Segments A-D), Segment A (GRLS) is the one that best explains the observed density while also fulfilling chemical constraints with a sensible hydrogen-bonding network and electrostatic interactions.

To obtain an unbiased, quantitative measure of how well each model with correct N-terminal to C-terminal directionality fits the map, we calculated per-residue real-space correlation coefficients (RSCC) between each model and the map, using the program phenix.validation_cryoem from the Phenix suite (Liebschner et al., 2019). The results are summarized in Table M1 below. RSCC rows are duplicated because density assigned to the ALC1 linker is present on both acidic patches of the nucleosome, and we modeled the peptide and analyzed the model fit into the density on both sides of the nucleosome (all figures above show chain K).

**Segment A sequence**	**G**	**<u>R</u>**	**S**	**L**
Segment A RSCC (chain K)	0.518	0.562	0.574	0.501
Segment A RSCC (chain L)	0.411	0.551	0.544	0.465
**Segment B sequence**	**L**	**<u>R</u>**	**N**	**K**
Segment B RSCC (chain K)	0.458	0.586	0.487	0.388
Segment B RSCC (chain L)	0.485	0.567	0.442	0.434
**Segment C sequence**	**K**	**<u>R</u>**	**K**	**R**
Segment C RSCC (chain K)	0.342	0.535	0.425	0.433
Segment C RSCC (chain L)	0.470	0.554	0.401	0.431
**Segment D sequence**	**K**	**<u>R</u>**	**V**	**L**
Segment D RSCC (chain K)	0.352	0.565	0.451	0.393
Segment D RSCC (chain L)	0.403	0.536	0.426	0.463

Table M1. RSSC values for each of the four residues of the ALC1 linker in each of the models with correct N-terminal to C-terminal directionality. The arginine residue occupying the "anchor" position is underlined.

While the arginine residue inserted into the acidic patch binding pocket exhibits high RSCC values regardless of the identity of the surrounding residues in the model (Table M1, column highlighted in dark grey), the model with a serine residue at the C-terminus of this arginine anchor shows a markedly higher RSCC at this location in

comparison to all the other models (Table M1, column highlighted in light grey). By this objective metric, Segment 1 (GRSL, as initially presented in our manuscript, highlighted in green in Table M1) is the one that best explains the observed density over all four residues spanned by the additional density.

To summarize, we modeled Segment A (GRSL) because it clearly best explains the observed density, both in terms of visual inspection of the experimental cryo-EM map and also quantitatively in terms of the per-residue real-space correlation coefficients.
